# From Gel to Crystal: Mechanism of HfO_2_ and ZrO_2_ Nanocrystal Synthesis in Benzyl Alcohol

**DOI:** 10.1021/jacs.4c00678

**Published:** 2024-04-08

**Authors:** Eline Goossens, Olivia Aalling-Frederiksen, Pieter Tack, Dietger Van den Eynden, Zarah Walsh-Korb, Kirsten M. Ø. Jensen, Klaartje De Buysser, Jonathan De Roo

**Affiliations:** † Department of Chemistry, Ghent University, 9000 Ghent, Belgium; ‡ Department of Chemistry, University of Basel, 4058 Basel, Switzerland; ¶ Department of Chemistry, University of Copenhagen, Copenhagen 2100, Denmark

## Abstract

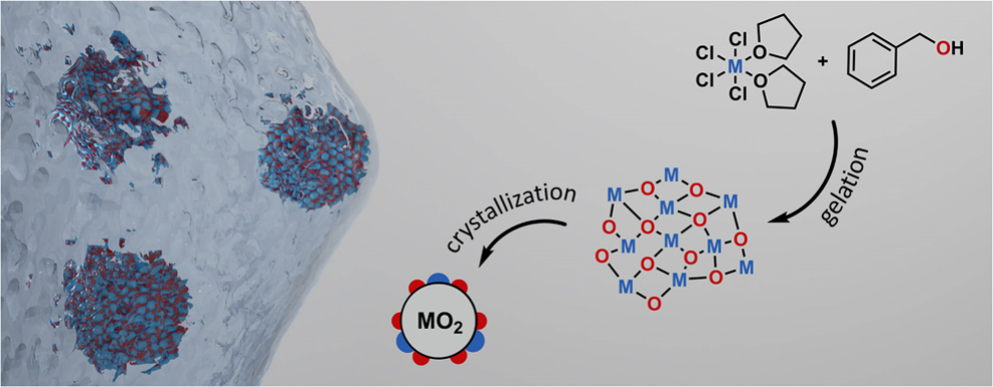

Nonaqueous sol–gel syntheses have been used to make many types of metal oxide nanocrystals. According to the current paradigm, nonaqueous syntheses have slow kinetics, thus favoring the thermodynamic (crystalline) product. Here we investigate the synthesis of hafnium (and zirconium) oxide nanocrystals from the metal chloride in benzyl alcohol. We follow the transition from precursor to nanocrystal through a combination of rheology, EXAFS, NMR, TEM, and X-ray total scattering (PDF analysis). Upon dissolving the metal chloride precursor, the exchange of chloride ligands for benzylalkoxide liberates HCl. The latter catalyzes the etherification of benzyl alcohol, eliminating water. During the temperature ramp to the reaction temperature (220 °C), sufficient water is produced to turn the reaction mixture into a macroscopic gel. Rheological analysis shows a network consisting of strong interactions with temperature-dependent restructuring. After a few minutes at the reaction temperature, crystalline particles emerge from the gel, and nucleation and growth are complete after 30 min. In contrast, 4 h are required to obtain the highest isolated yield, which we attribute to the slow *in situ* formation of water (the extraction solvent). We used our mechanistic insights to optimize the synthesis, achieving high isolated yields with a reduced reaction time. Our results oppose the idea that nonaqueous sol–gel syntheses necessarily form crystalline products in one step, without a transient, amorphous gel state.

## Introduction

Sol–gel processing is a rich and historic field of materials science.^1,2^ It is used to make silicate and non-silicate oxides in the form of coatings, powders, porous monoliths, etc. The basis of sol–gel chemistry is the controlled hydrolysis of a metal precursor;^2−4^ the addition of water generates a hydroxide species (M–OH). Hydrolysis is followed by condensation, leading to M–O–M bonds.
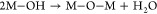
1In the case of silica, first, a dispersion of small particles (a sol) is formed. Further aggregation and condensation results in a nonfluid 3D network (a gel). Sol–gel processing is also highly valuable to make multimetal ceramics^5,6^ since it ensures intimate mixing of the metals at the atomic level, in contrast to traditional solid-state chemistry where powders react.

For high-valence transition metals (e.g., Ti(IV) or Nb(V)), hydrolysis and condensation reactions in water occur extremely fast and almost simultaneously. The resulting amorphous oxide product requires a postsynthetic heat treatment to obtain the crystalline phase.^7^ To slow down the reaction rate, nonaqueous processes were developed.^8−10^ In the latter, condensation could take place via organic reactions, for example by alkyl halide elimination.^11,12^
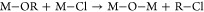
2Initially, the products were still largely amorphous powders and gels. However, with the introduction of benzyl alcohol as solvent and oxygen donor, crystalline nanoparticles were obtained.^13^ Titania nanocrystals were synthesized at 40 °C from TiCl_4_ and benzyl alcohol, albeit with long reaction times of 7–14 days. Benzyl alcohol turned out to be a highly versatile solvent and allowed to synthesize many metal oxide nanocrystals.^14−18^ One hypothesis as to why benzyl alcohol delivers crystalline nanoparticles is that the condensation reaction is so slow that the thermodynamic product (the crystal) is directly formed instead of the kinetic product (the amorphous gel).

The most frequently used precursors in nonaqueous syntheses of group 4 oxide nanocrystals are the metal alkoxides (M(OR)_4_) and metal halides (MX_4_, X = F, Cl, Br, and I).^18^ When dissolving TiCl_4_ in an excess of ethanol, partial ligand exchange takes place, forming TiCl_2_(OEt)_2_ and HCl; see eq 3.^19^ One equivalent of alcohol further coordinates to the diethoxydichlorotitanium complex. For ZrCl_4_ both a single and double exchange takes place; see eq 4.^20^ Although not well described, the extent of exchange also depends on the type of alcohol used. Based on the comparable experimentally determined equilibrium constants of HfCl_4_ to ZrCl_4_, it is expected that HfCl_4_ follows eq 4 as well.^21^ Thorium chloride coordinates 4 equiv of methanol, ethanol, or isopropanol, and no substitution takes place (eq 5).^22^ ThCl_4_ does react with *tert*-amyl alcohol, releasing alkyl chloride and the E1 elimination product (alkene).^22^
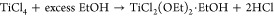
3
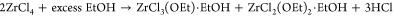
4
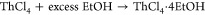
5

The organic pathways, responsible for the condensation reactions, have been elucidated and include esterification, etherification, halide elimination, etc.^12^ The nucleation and growth mechanism, however, are not fully understood. When mixing TiCl_4_ and benzyl alcohol at 85 °C, X-ray scattering studies have shown that TiO_2_ nanocrystals form suddenly after an induction time of 60 min.^24^ The particles are crystalline from their formation and aggregate into precipitates. When mixing Ti(OiPr)_4_ and benzyl alcohol at 175 °C, again an induction time was observed, after which the pressure quickly increased.^25^ The effect is ascribed to the catalytic formation of water and coincides with the formation of nanocrystals. The *in situ* formation of water has also been reported in the reaction of HfCl_4_ in benzyl alcohol.^26^ Presumably, the chloride to alkoxide exchange releases HCl, which catalyzes etherification. Similar chloride to alkoxide exchange was also reported for niobium chloride in benzyl alcohol.^27^ Detailed pair distribution function (PDF) analysis showed polymeric species formed from partial condensation of niobium chloride alkoxide complexes. *In situ* water formation can transform tetragonal ZrO_2_ nanocrystals into the monoclinic crystal phase.^28−30^ Neutral precursors such as zirconium isopropoxide, ethoxide, and acetate react in benzyl alcohol to give cubic/tetragonal ZrO_2_, while zirconium chloride yields monoclinic ZrO_2_.^31^ In the presence of trifluoroacetic acid (TFA), zirconium isopropoxide in benzyl alcohol yields the monoclinic structure.^31^ The strong acid catalyzes etherification and induces *in situ* water formation. While ZrO_2_ thus exhibits rich polymorphism, hafnium isopropoxide, *tert*-butoxide, ethoxide, and chloride all yield monoclinic HfO_2_ in benzyl alcohol.^18^ Cubic HfO_2_ is obtained when doping HfO_2_ with lanthanides (from 8% doping on)^32^ or when reacting hafnium *tert*-butoxide in benzylamine.^33^

Hafnium oxide nanocrystals are particularly interesting since they are relevant for memory devices,^34−36^ as X-ray CT contrast agents,^37−42^ for scintillators,^32,43,44^ for catalysis,^45^ or for radiotherapy.^46^ While HfO_2_ can be synthesized in benzyl alcohol from HfCl_4_ in a pressure bomb (autoclave),^47^ the reaction can also be performed in a microwave reactor, considerably shortening the reaction time to 3–4 h.^26^ We recently scaled up this reaction to deliver multiple grams of HfO_2_ per batch.^38^ Key to the scale-up is the use of the highly soluble and less reactive HfCl_4_·2THF complex, allowing for a high precursor concentration in benzyl alcohol. After synthesis, HfO_2_ nanocrystals can be deaggregated in nonpolar solvents by functionalization with fatty acids, in the presence of a base.^48^ Tight binding of nitrodopamine derivatives render HfO_2_ nanocrystals stable in aqueous buffer solutions.^49^

Given the importance of HfO_2_, we study here its synthesis from the most economical precursor: hafnium chloride. We find a surprising gel intermediate that recrystallizes to form HfO_2_ nanocrystals. EXAFS shows a gradual transition in the first coordination shell of hafnium from chloride to oxygen during the heating ramp. This transition is related to water formed in the reaction, mainly due to acid-catalyzed etherification. Several control experiments with water scavengers and alkoxide precursors confirm this hypothesis. Similar results are obtained for the synthesis of ZrO_2_ nanocrystals from zirconium chloride. The gel consists of a strong 3D network and aggregated nanoparticles with structural coherence up to 1 nm, as confirmed by *in situ* rheology and X-ray total scattering experiments with PDF analysis. The latter also shows that crystallization proceeds quickly. Using the mechanistic insights, we could further reduce the reaction time to 1 h while obtaining excellent isolation yields. Our results defy the hypothesis that a gel should always be avoided to obtain crystalline nanoparticles in solution.

## Results and Discussion

### Appearance of a Gel Phase

**Figure 1:**
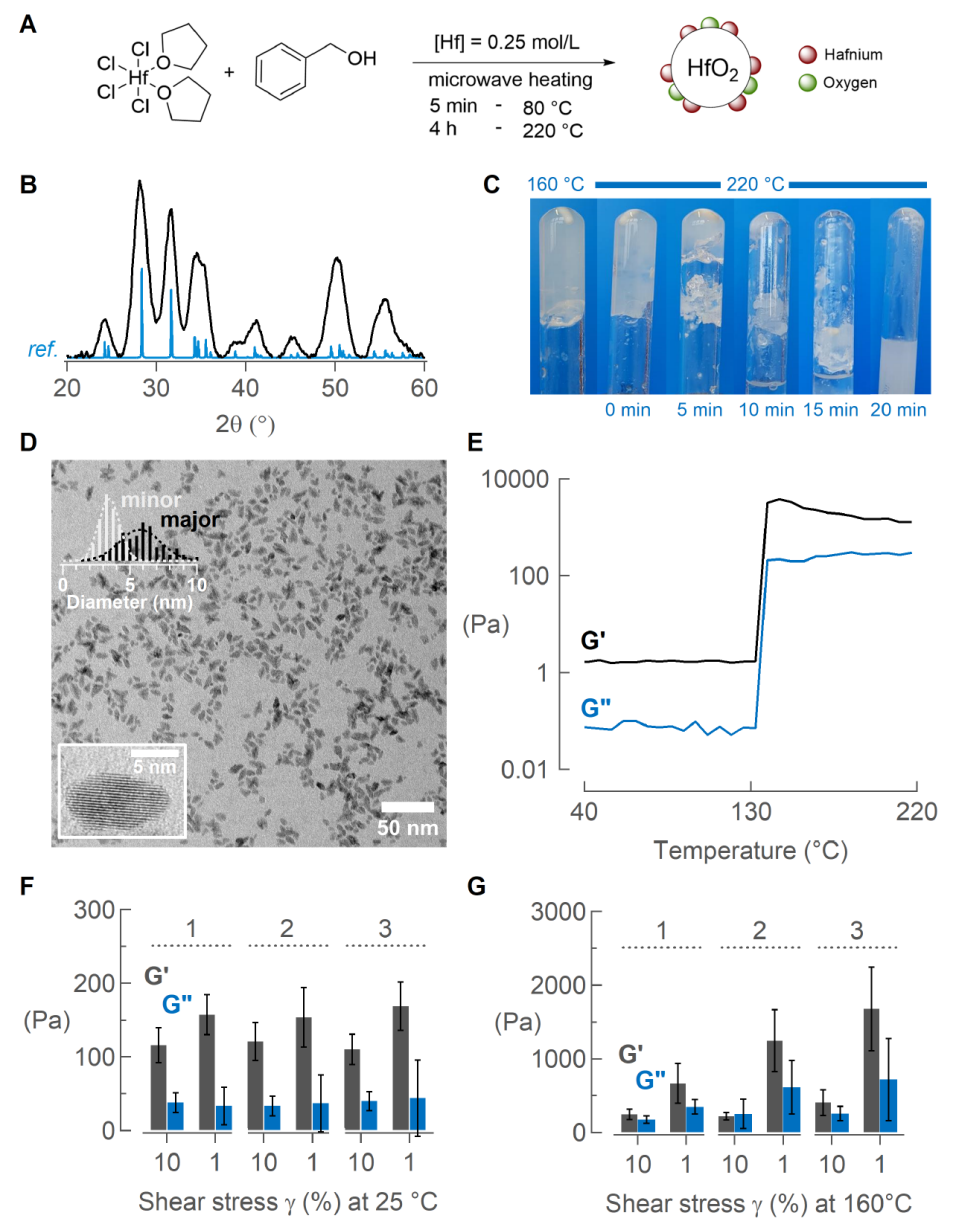
(A) Microwave-assisted solvothermal synthesis of HfO_2_ nanocrystals starting from HfCl_4_·2THF in benzyl alcohol. (B) XRD spectrum (black) with a monoclinic reference spectrum (blue, Crystallographic Open Database ID 9013470). (C) Pictures of the upside down microwave tube, stopped at different time points in the reaction to indicate the disappearance of the gel over time. (D) TEM image of the synthesized NCs shows their ellipsoidal shape. The size distribution and a zoom of a single NC are shown in the top and bottom left corner, respectively. (E) The rapid increase in storage modulus *G*′ and loss modulus *G*″ over time (average of 3 measurements) indicates the point where the solution turns into a gel. (F) Storage modulus *G*′ and loss modulus *G*″ of the gel after repeatedly (three cycles) applying 10% (stress) and 1% (recovery) shear stress (γ) at 25 °C and (G) at 160 °C.

We start from the optimized hafnia synthesis using HfCl_4_·2THF in benzyl alcohol. The hafnium precursor dissolves readily and is heated first to 80 °C and subsequently to 220 °C in a closed microwave vessel (Figure 1A). The synthesis yields hafnium oxide nanocrystals featuring a monoclinic (*P*2_1_/*c*) crystal structure, evidenced by powder X-ray diffraction (XRD) analysis (Figure 1B). The nanocrystals are ellipsoidal, with a major axis of 6.2 ± 4.8 nm and a minor axis of 4.0 ± 2.4 nm (μ ± 3σ) as determined by transmission electron microscopy (TEM) (Figure 1D). With the use of a camera in the microwave chamber, we observe a phase of high viscosity during the heating ramp (see the video supplied as Supporting Information). Over the course of 20 min, at a reaction temperature of 220 °C, the solution returns to a normal liquid state. The same observations can be made from samples that are quickly quenched to room temperature at different time points in the reaction; see Figure 1C (the microwave vessels are positioned upside down). From 160 °C and continuing up to 5 min at 220 °C, the reaction mixture gelled, defying gravity. The gel gradually liquefies as the reaction continues.

We characterize the viscoelastic behavior more in depth via *in situ* rheology (Figure 1E). The elastic behavior is described by the storage modulus *G*′, representing the stored deformation energy, i.e., how much energy has to be put into a sample to distort it. The viscous behavior is described by the loss modulus *G*″, which characterizes the deformation energy that is dissipated into heat when the material is put under shear stress. Both *G*′ and *G*″ rapidly increase between 130 and 150 °C. This is correlated to a rapid increase in viscosity as the mixture turns into a gel. In a typical gel transition, the mixture is viscous (*G*′ < *G*″) before it reaches the gelpoint, defined by the crossover of the two curves (*G*′ = *G*″). What follows is increasingly elastic behavior (*G*′ > *G*″). However, the mixture of HfCl_4_·2THF in benzyl alcohol at room temperature already exhibits *G*′ > *G*″ (Figure 1E), in fact benzyl alcohol also features *G*′ > *G*″ (Figure S1A). This is not surprising, as the benzyl rings in benzyl alcohol can physically interact with one another through π–π stacking, contributing to the viscosity of the solvent (5.84 mPa.s at 25 °C). Given that the solvent exhibits a *G*′ greater than *G*″ from the start, the technical gel-point in the formation of the HfO_2_ gel is absent, but it is clear that a transformation takes place between 130 and 150 °C. To understand the nature of the gel network, in particular, if there were physical or chemical interactions creating a 3D structure between the inorganic polymer chains or if the system was randomly stacked with no specific interactions, we examined the recovery behavior of the system after the application of shear stress. This involved applying a 10% shear stress at 1 Hz for 10 s, followed by monitoring the system for 60 s at 1% shear stress and repeating this cycle three times. Figure 1F,G shows the recovery measured at 25 and 160 °C. Note that this was performed on a gel formed by microwave heating that had been quenched to room temperature and re-equilibrated at 25 or 160 °C. At 25 °C the gel phase remains largely stable and recovers completely after every high shear application, confirming the presence of strong intermolecular interactions within the network. Furthermore, the network is only mildly disturbed at 10% shear, suggesting the network could withstand greater deformations. Interestingly, at 160 °C the gel phase becomes increasingly stronger with each application of high shear. We hypothesize that this is an effect of the re-equilibration at 160 °C, and that this temperature-dependent structuring leads to further condensation of the amorphous network with an increasing amount of covalent bonds that contribute to increasing elastic behavior of the network. At room temperature, no additional condensation can take place. The *G*′ and *G*″ curves at both temperatures are plotted in Figure S1B.

Similar gel behavior is observed across different chloride precursors HfCl_4_·2THF, HfCl_4_, ZrCl_4_·2THF, and ZrCl_4_. *In situ* rheology reveals that the gel point occurs at a slightly higher temperature for the zirconium precursor (Figure S1C). Depending on the heating ramp, the observed gel points can shift by 10 °C (Figure S1D). For a slower heating rate (0.01 °C/s), the transition to gel formation occurs at higher temperature (approximately 130 °C) and reaches higher storage and loss modulus values. In fact, *G*′ and *G*″ are an order of magnitude greater than those achieved at a faster heating rate (0.03 °C/s), in which the gel transition begins at 120 °C. This behavior follows the assumption that there is temperature-dependent and time-dependent (i.e., kinetically controlled) structuring/restructuring present. Increasing the equilibration time (i.e., slower heating rate) allows for more covalent bonds to be formed, resulting in a stronger gel.

### Precursor Structure

To understand the origin of this gel formation, we take a step back to examine the actual precursor, i.e., the hafnium species that is formed at room temperature after dissolving the HfCl_4_·2THF complex. Based on eqs 3–5, a halide to alkoxide exchange is expected. The ratio of chloride to alkoxide can be assessed by complexation with a Lewis base; tri-*n*-octylphosphine oxide (TOPO). The ^31^P NMR chemical shift is sensitive to the Lewis acidity of the metal complex, which decreases in the series:^50^
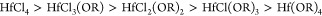
6

**Figure 2:**
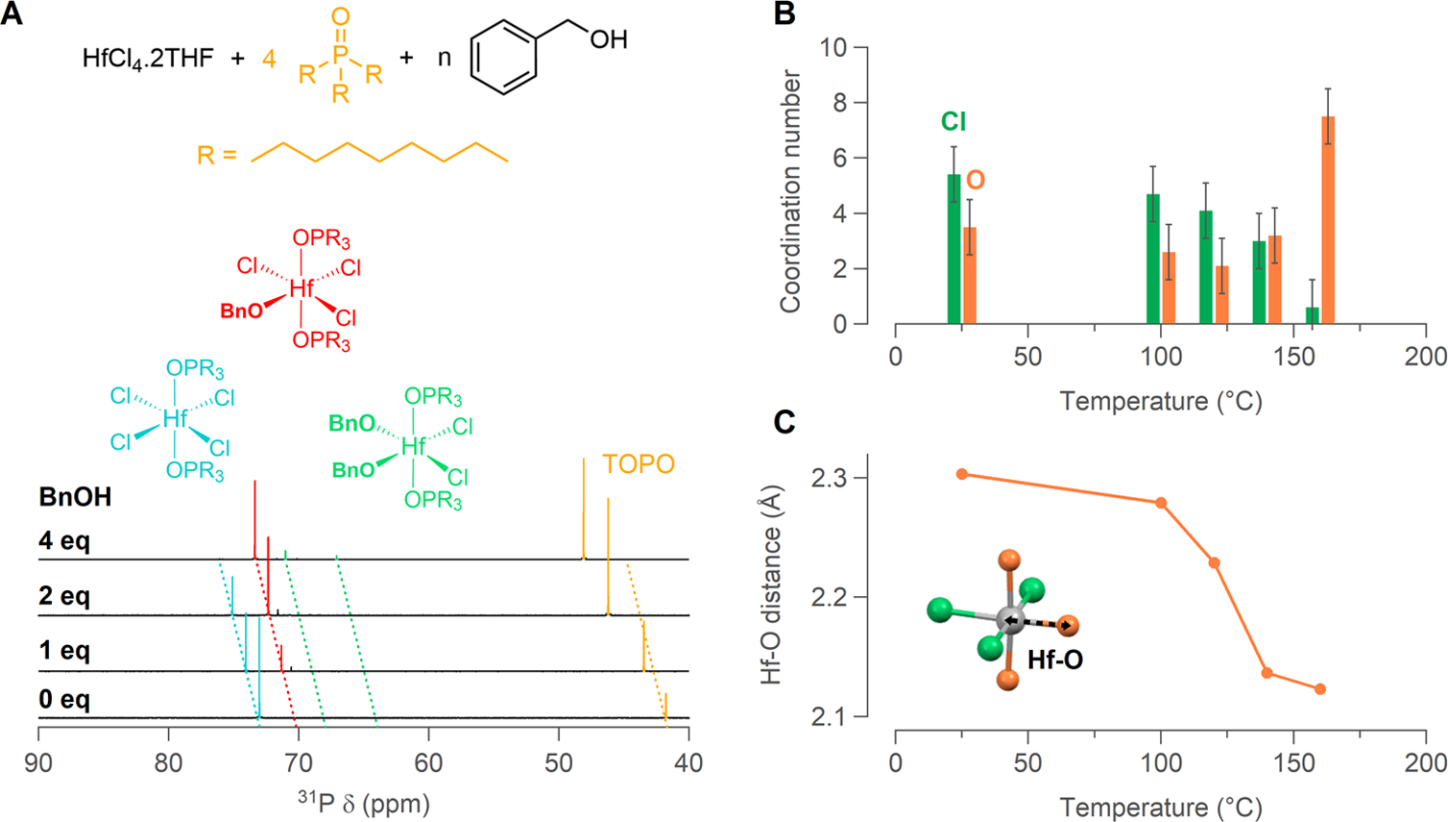
(A) Titration of HfCl_4_·2THF with benzyl alcohol in the presence of TOPO in C_6_D_6_, followed via ^31^P NMR. The spectra have a relative *x*-offset of 1 ppm with respect to each other for clarity. (B) Coordination numbers of chloride and oxygen surrounding the hafnium center, calculated from the EXAFS data. (C) Hf–O distance contraction at increasing temperature. The octahedral of the HfCl_3_OBn·2THF structure is shown for clarity.

We first react HfCl_4_·2THF with different equivalents of benzyl alcohol, remove the volatile HCl, and subsequently add TOPO. The ^31^P NMR spectra are shown in Figure 2A. After adding 1 equiv, we observe both the tetrachloride species detected at 73 ppm and the trichloride at 69 ppm. Upon adding more benzyl alcohol, the amount of HfCl_3_(OBn) increases, and after adding 4 equiv no HfCl_4_ is observed anymore. Zirconium chloride appears slightly more reactive as the trichloride is more intense at 1 and 2 equiv of benzyl alcohol (Figure S4A). These results are consistent with those in eq 4.

### From Precursor to Gel

We used extended absorption fine structure (EXAFS) spectroscopy to further investigate the coordination environment of hafnium at room temperature and during the heating ramp (Figure 2B,C). At room temperature, the EXAFS data are well-described by a combination of Hf–O and Hf–Cl scatter paths (see Figure S3 for the fits). The determined Hf–O distance is 2.30 Å and the Hf–Cl distance is 2.43 Å. For comparison, the crystal structure of *cis*-HfCl_4_·2THF features the Hf–O distance at 2.2 Å and the Hf–Cl distance at 2.36–2.39 Å.^51^ During heating of the reaction mixture in a three-neck round-bottom flask, we took aliquots at different temperatures and analyzed each with EXAFS; see Figure 2B,C. With temperature, the amount of Hf–Cl scatterers decreases, and especially around the gelation temperature, the amount of oxygen scatterers increases. This suggests a further exchange of chloride for alkoxide (or hydroxide) upon heating and a drastic change of the coordination shell at the gel point, as chloride has almost completely disappeared from the coordination shell at 160 °C. Simultaneously, the Hf–O distance decreases from 2.30 to 2.12 Å, pointing toward a further structural rearrangement such as the formation of M–O–M bridges. All refined parameters, bond distances, and coordination numbers are reported in Table S2. Similar results were obtained for zirconium (Figure S4B and Table S2), although we did not observe the same almost complete elimination of chloride from the coordination shell at 160 °C. This is consistent with the higher gelation temperature seen in rheology for zirconium.

We hypothesize that water is the main driver for releasing chloride from the coordination shell, enabling further condensation. Water can be formed by alcohol-to-ether conversion, which is readily catalyzed by strong acids, such as HCl:^52^
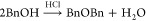
7A self-sustaining pathway can be conceived. Small amounts of HCl are liberated upon the dissolution of hafnium chloride in benzyl alcohol. During the heating ramp, HCl catalyzes the etherification, releasing water. Water reacts with the hafnium species (e.g., HfCl_3_OBn), forming hafnium hydroxide moieties and releasing more HCl. A higher amount of catalyst (HCl) increases the rate of water formation, which increases the rate of HCl liberation. One can write this autocatalysis concisely:
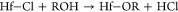
8
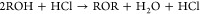
9
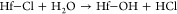
10

**Figure 3:**
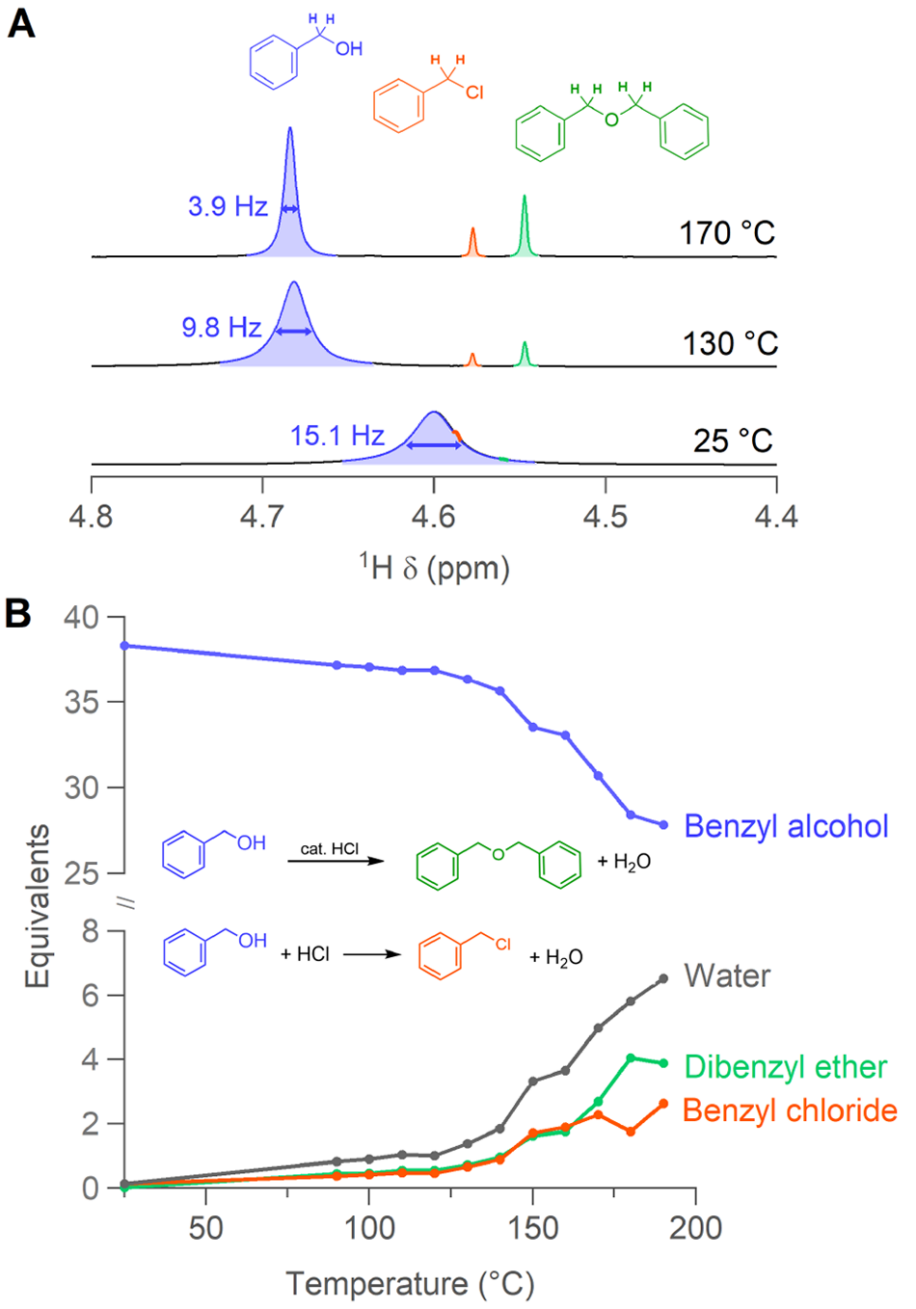
(A) ^1^H NMR assignment of the resonances in the supernatant between 4.4 and 4.8 ppm. The full width at half-maximum (in Hz) of the benzyl alcohol peak is indicated and decreases with increasing temperature. (B) Equivalents of benzyl alcohol, dibenzyl ether, and benzyl chloride with respect to hafnium (taking into account other side products present) in the reaction supernatant at increasing temperature. The amount of water is calculated based on the amount of dibenzyl ether and benzyl chloride detected (calculations in Supporting Information).

To test this hypothesis, the formation of dibenzyl ether and other side products was tracked with ^1^H NMR spectroscopy by taking aliquots from a solution of HfCl_4_·2THF in benzyl alcohol (heated in a three-neck round-bottom flask). The ^1^H NMR spectrum between 4.4 and 4.8 ppm is of special interest since the CH_2_ peaks of BnOH and BnOBn are distinguished (Figure 3A). We also observe a third resonance, which we assign to benzyl chloride (BnCl). BnCl is formed from the reaction of HCl with BnOH and also liberates water as a byproduct (see Figure S5 for a control experiment with HCl and BnOH). Interestingly, both BnOBn and BnCl are present even from the start of the reaction at 25 °C (Figure 3A), albeit in very low concentration (both <0.15 equiv with respect to Hf). Indeed, some HCl has been formed at room temperature; see Figure 2. With increasing temperature, the concentration of BnOBn and BnCl increases and thus also the water concentration increases. The benzyl alcohol resonance becomes narrower with temperature, which could be due to several factors such as a different alkoxide to alcohol equilibrium, different pH, or different water content. At 150 °C the total amount of water generated is 3.3 equiv with respect to hafnium. When the gel point (160–170 °C) is reached, 5.0 equiv of water are formed. Similar kinetics were observed for microwave reactions that were quenched to room temperature for sampling (Figure S6). After 30 min at 220 °C, the etherification reaction reaches equilibrium with 14.6 ± 0.2 (μ ± σ) equiv of dibenzyl ether formed. The benzyl chloride concentration increases more moderately throughout the reaction and reaches a maximum of 3.5 ± 0.3 equiv. From the NMR data we conclude that between 4 and 5 equiv of water with respect to hafnium are present when the gel network is formed. When only 4 equiv of benzyl alcohol are used (and using benzyl ether as the solvent), also a gel is formed. For ZrO_2_ we observe a similar trend (Figure S7). The amount of water builds up slightly more slowly in solution. At 170 °C, 4.0 equiv of water with respect to zirconium are present and 5.1 equiv is only reached at 190 °C, which is consistent with the slower gelation observed in rheology measurements. For SiO_2_ it has been found that a minimum of 1 equiv of water is required to produce chain-like polymers.^53^ However, the gelation time is shortest for 2–4 equiv.^54^

In control experiments with Hf(OiPr)_4_.iPrOH and Hf(OtBu)_4_ no gel intermediate was observed in a time frame of 30 min at 230 °C. As there is no strong acid present to catalyze the condensation of BnOH, the concentration of BnOBn ether remains low (3.6 equiv after 96 h at 220 °C). When anhydrous TFA is added to the reaction mixture, a gel is observed either after reaching 200 °C for Hf(OtBu)_4_, or after 15 min at 220 °C for Hf(OiPr)_4_.iPrOH. The intense resonance of BnOBn in the ^1^H NMR spectrum confirms the etherification pathway (Figure S8A). The final nanocrystal products are different compared to the products from the chloride precusors. The addition of TFA resulted in aggregated particles and amorphous products (Figure S8B). Previous research has demonstrated that adding TFA to Zr(OiPr)_4_·iPrOH causes a switch from cubic to the more stable monoclinic ZrO_2_ NCs.^31^ It was hypothesized to result from a different reaction mechanism, although the occurrence of the gel phase was not observed as the research was done using an autoclave (i.e., a black box). Gel formation can be caused by any reaction that produces water *in situ*. For example, the addition of acetic acid to a reaction mixture with Hf(OiPr)_4_·iPrOH also causes gelation as acetic acid reacts with benzyl alcohol to form the benzyl acetate ester and water (Figure S8A).

**Scheme 1:**
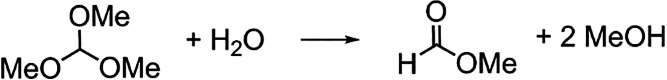
Reaction Mechanism of Trimethyl Orthoformate with Water to Methyl Formate and Methanol

If the gel formation is indeed caused by *in situ* water formation, then we should be able to avoid it by removing produced water from the reaction mixture. We identified trimethyl orthoformate (TMOF) as an orthogonal water scavenger, which reacts fast and irreversibly with water to methyl formate and methanol, see Scheme 1.^55^ Indeed, after adding >5 equiv of TMOF to the reaction mixture with HfCl_4_·2THF, we observe the gel phase only after half an hour at 220 °C, even though the BnOBn and BnCl resonances are still clearly observed in the ^1^H NMR spectrum. In addition, resonances pertaining to methyl formate and methanol are also detected (Figure S9A). Interestingly, delaying the gel intermediate had little effect on the final products, although they are somewhat more aggregated (Figure S9B).

### From Gel to Nanocrystals

To gain insight in the transformation from gel to crystals, we turn to *in situ* X-ray total scattering combined with pair distribution function analysis (PDF), a technique that has proven very effective to understand formation mechanisms.^27,56−59^ HfCl_4_·2THF, dissolved in BnOH, is loaded in a 0.7 mm glass capillary. The reaction mixture is heated to the gel point (150 °C) and after 26 min the temperature is increased to 220 °C.

**Figure 4:**
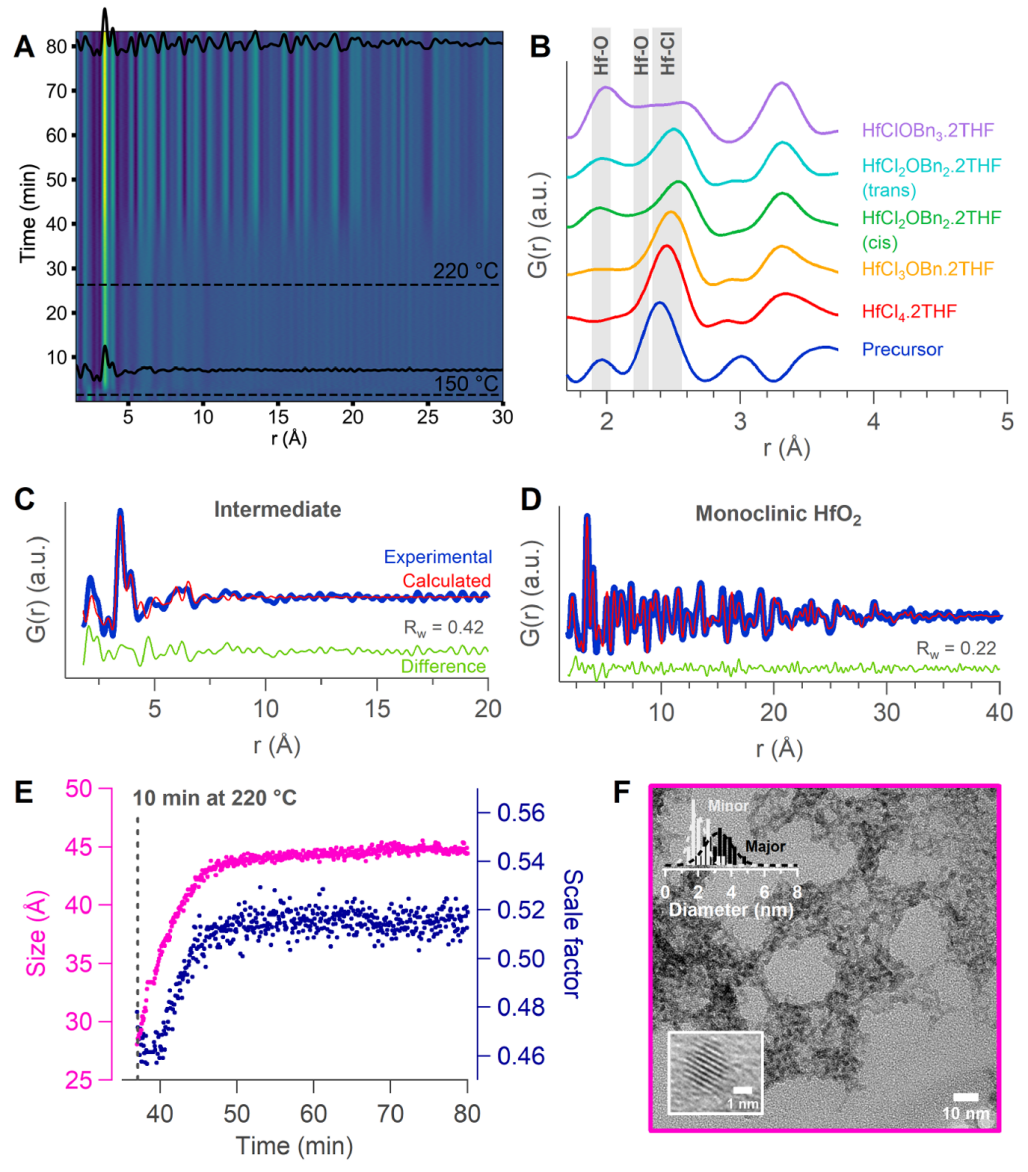
(A) *In situ* total scattering data showing *G*(*r*) as a function of time. Dashed lines indicate when temperature is increased. Selected PDFs from the intermediate and the final stage of the reaction are plotted. (B) PDF of the precursor structure with major peaks assigned and in comparison to simulated PDFs based on the possible precursor structures. Fit of the PDF data of (C) the intermediate gel, collected after 9 min of reaction at 150 °C, and (D) the final product to m-HfO_2_, collected after 81 min of total reaction time. (E) Refined crystallite size and scale factor as a function of time. (F) TEM image of a sample after 3 min at 220 °C. The size distribution (25 particles) and zoom of a single NC are shown in the top and bottom left corner, respectively.

A contour plot of the reduced PDFs (G(r)) as a function of time is shown in Figure 4A. At room temperature, the PDF of the precursor shows a major peak at 2.4 Å (Figure 4B), which is assigned to the Hf–Cl distance (consistent with the EXAFS data). As expected for a monomeric species, no higher correlations are detected (Figure S10B). Previously, PDF measurements of HfCl_4_ in methanol, did show higher correlations, consistent with a mixture of different structures ranging from monomers to trimers.^60^ In contrast, ZrCl_4_ exists as a monomer in methanol or ethanol and is octahedrally coordinated. The coordination shell consists of chloride ions and solvent molecules. This points again to the higher condensation propensity of hafnium.^23^ Due to the weak scattering and challenging removal of the benzyl alcohol background, we did not fit the precursor PDF. Via DFT calculations, we obtained the structures of the possible precursor complexes: HfCl_4–*x*_(OBn)_*x*_·2THF) with *x* = 0–3 (Figure S12, xyz files supplied in Supporting Information). To reduce the computational cost, we performed the calculations with Zr and assumed equivalent structures for hafnium. Interestingly, the alkoxide features a shorter Zr–O bond length (1.95–2.00 Å) compared to the neutral Lewis base (Zr–O; 2.20–2.27 Å, bond lengths are indicated in Figure S12). Using the computed structures, we simulated the corresponding PDFs (Figure 4B). The first coordination sphere (1.8–2.8 Å) of the experimental precursor PDF shows the most similarities with the calculated PDFs of hafnium trichloride benzyloxide and hafnium dichloride dibenzyloxide, consistent with eq 4.

At 150 °C the gel intermediate appears, as is apparent by the increase in oxygen in the coordination shell, consistent with the EXAFS data. We find structural features in the PDF between 2 and 10 Å, indicating condensation into M–O–M bonds; see Figure 4C. The local correlations related to the final monoclinic structure are already present (Hf–O = 2.1 Å, Hf–Hf (edge-sharing) = 3.5 Å, and Hf–Hf (corner-sharing) = 4.0 Å). The PDF can indeed be refined using the monoclinic HfO_2_ crystal structure with space group *P*2_1_/*c*.^61^ All refined parameters are presented in Table S3. The refined crystal size appeared to be 10 Å. Given the data from rheology, we propose that these small units build up larger disordered structures. The nanoscale signature of the intermediate is similar to the one previously reported from the solvothermal syntheses of zirconia.^62^

As the temperature is further increased to 220 °C, the crystallite size starts growing, as indicated by the appearance of long-range order, observed in Figure 4A. The final PDF pattern is again refined using the monoclinic HfO_2_ crystal structure (Figure 4D). The refined crystallite size is 44 Å, consistent with the minor axis of the ellipsoidal particles according to TEM. To further elucidate the transformation from gel to crystals, we do sequential refinements starting from the final product in the backward direction until the gel phase. Excellent refinements are obtained between 10 and 80 min at 220 °C (Figure S11B). The refined crystallite size increases quickly after reaching 220 °C and stabilizes after 24 min at 220 °C, Figures 4E and S11A. The unit cell contracts with increasing crystallite size, as demonstrated from the refined unit cell volume as a function of time (Figure S10C). The crystallization yield is approximated both by the refined scale factor (Figure 4E) and by the integrated (111) peak in reciprocal space (Figure S11A). The yield saturates together with the crystal growth, indicating the end of the crystallization process and, thus, no further ripening.

The gel does not turn fully liquid until at least 20 min at 220 °C. Notwithstanding, we observe already crystalline nanoparticles in the gel after 3 min at 220 °C in TEM, Figure 4F. They appear highly aggregated and form a network. Consistent with the PDF data, they are smaller than the final NC size and can be described by a major axis of 3.4 ± 2.3 nm and a minor axis of 2.4 ± 1.6 nm (μ ± 3σ). Note that due to the high degree of agglomeration, the size distribution is made up of only 25 particles that could be individually resolved. After 10 min, we can extract and stabilize nanocrystals from the liquid phase. These nanocrystals already have their final size and do not grow anymore during further heating (Figure S13).

### Enhancing the Isolated Yield Using Mechanistic Insight

**Figure 5:**
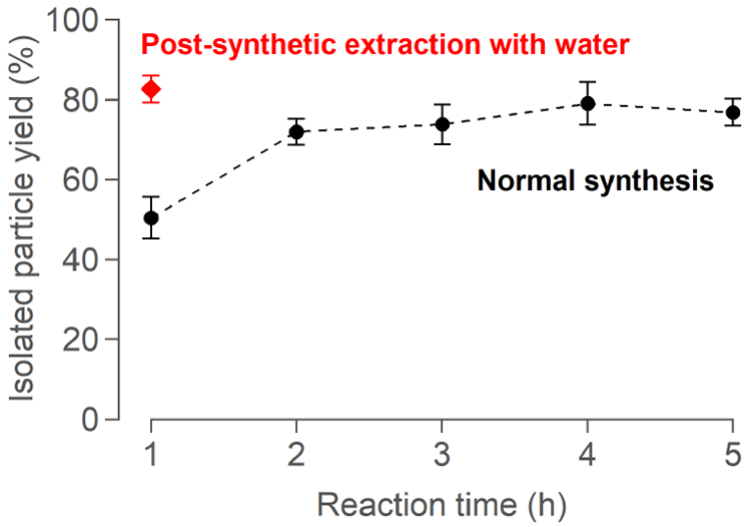
Isolated particle yield of HfO_2_ NC syntheses run for different reaction times. The yield can be significantly improved at only 1 h by adding water postsynthesis. Each reaction is performed in triplicate, and yield is determined gravimetrically.

We stopped the microwave synthesis at various time points, isolated and surface-functionalized the nanocrystals, and finally weighed the particles to gravimetrically determine the isolated yield of the functionalized particles; see Figure 5 and Table S4. Below 1 h, the yield is not reproducible due to the presence of the gel. After 1 h, the yield is only 50 ± 5% (μ ± σ). It increases to 72 ± 3% at 2 h or 77 ± 3% at 5 h. As the *in situ* PDF measurements indicate that nucleation and growth are complete after ∼30 min, it could seem surprising that the isolated yield increases over several hours.

The amount of water formed *in situ* builds up over the course of the reaction (see above). After synthesis, the HfO_2_ NCs are stabilized in this water phase, which is phase separated from the organic phase.^48^ We thus hypothesize that after 1 h the final number of particles has been reached, but the amount of water created is insufficient to stabilize all particles. As the reaction continues, more water is created, resulting in the extraction of more particles from the organic phase. We can drive this extraction equilibrium forward by adding water postsynthesis to the reaction mixture. Indeed, our isolated yield drastically improves at 1 h, reaching 83 ± 3%. By doing the extraction with water postsynthesis, we can therefore reduce the reaction time while even slightly improving the maximum yield.

## Discussion

The sol–gel route has evolved into an established method to synthesize oxide nanocrystals with a broad range of sizes, shapes, and compositions.^33,63−67^ The success of nonaqueous sol–gel routes compared to their aqueous counterpart is often accredited to their slower reaction rate as a consequence of the moderate reactivity of the C–O bond,^12^ allowing crystals (the thermodynamic product) to be formed immediately instead of an amorphous gel (the kinetic product). Amorphous products typically require high-temperature calcination to crystallize. Here, we showed that (i) even in nonaqueous syntheses, a gel can be formed when excess water is quickly formed *in situ* and (ii) the gel can recrystallize in solution to nanocrystals. Note that the structure of the amorphous product is likely very different in water or benzyl alcohol. Indeed, most aqueous procedures yield an amorphous precipitate and not a gel that traps the entire solvent.^68−71^ Consequently, the resulting gel in benzyl alcohol is less dense than the amorphous product in water, and it can be more easily restructured. In addition, the higher boiling point of benzyl alcohol allows for higher reaction temperatures and thus overcomes higher activation energies.

It is also often postulated that a nanocrystal precursor (P) converts into a monomer (M).^72,73^ Here, we observe the conversion of the precursor into a gel. While the exact mechanism by which the gel crystallizes into the nanocrystals remains unclear, we can exclude a LaMer mechanism, and consequently, the derived mass balance by Sugimoto is not valid in this case.^73,74^ Our results agree with other reports of nonclassical pathways to (oxide) nanocrystals, featuring disordered intermediates.^75−79^ However, such previous reports did not report a macroscopic gel phase. The amorphous intermediates were identified as nanoparticles, and the reaction mixture remains liquid. Interestingly, a gel phase was visually observed for the surfactant assisted synthesis of hafnium oxide nanocrystals from hafnium trifluoroacetate and oleylamine.^43^ The reaction of niobium chloride with benzyl alcohol also showed polymeric species according to PDF analysis, although no macroscopic observations were mentioned.^27^ This may indicate a more general nature of our findings, and the question arises if other (metal oxide) syntheses also go through an amorphous intermediate or gel phase. It may easily go unnoticed since most metal oxide syntheses need pressurized vessels, which are often literally a black box. The presence of a macroscopic gel will depend on the precise synthetic conditions (kinetics and extent of water formation) but also on the metal. The early transition metals are more prone to hydrolysis and condensation compared to the late transition metals and thus more readily form gels.^80^ Further theoretical work should focus on building a framework for the recrystallization of nanocrystals from amorphous structures.

## Conclusion

We presented new insights into the mechanism of the HfO_2_ and ZrO_2_ NC syntheses starting from the metal chloride in benzyl alcohol. Upon dissolution of the metal chloride, partial ligand exchange of chloride for benzyloxide takes place. The HCl byproduct catalyzes the etherification of benzyl alcohol, thus producing water. Water hydrolyzes the metal alkoxychloride complex further, releasing even more HCl. These reactions happen during the heating ramp when the reaction mixture is brought to 220 °C. Around 160 °C, 4 equiv of water is formed, which is enough to completely hydrolyze the metal, and condensation reactions take place. The short-range structure can be described by a mixture of edge- and corner-sharing polyhedra, while the macroscopic structure is a gel with strong intermolecular interactions. At 200 °C, the gel rapidly recrystallizes (in 30 min) into mature nanocrystals. However, the isolated yield depends on the amount of water produced (or added) to extract the protonated particles. Our results present a new view on sol–gel syntheses, rebutting the idea that a gel needs to be avoided for the production of crystalline particles.

## Experimental Section

### Materials

Hafnium(IV) chloride (98%), zirconium(IV) chloride (≥99.5%), oleic acid (90%), oleylamine (70%), trimethyl orthoformate (99%), trifluoroacetic acid (99%), acetic acid (≥99%), dibenzyl ether (98%), and benzyl chloride (99%) are obtained from Sigma-Aldrich. Benzyl alcohol was purchased either anydrous (99.8%) or as ReagentPlus (≥99%) from Sigma-Aldrich; the latter was then vacuum distilled and stored over sieves. Tetrahydrofuran (extra dry, 99.5%) was purchased from Acros Organics. Hydrochloric acid (≥37%) was purchased from Chemlab. Tri-*n*-octylphosphine oxide (99%) was puchased from Strem chemicals and recrystallized according to Owen et al.^81^ Solvents used for synthesis were purchased from Chemlab or Sigma-Aldrich. Deuterated solvents (CDCl_3_ and C_6_D_6_) were purchased from Sigma-Aldrich or Eurisotop.

All manipulations were performed in air, unless otherwise indicated. All chemicals are used as received unless otherwise mentioned. When required, organic solvents are dried according to the procedure described by Williams et al.^82^ making use of 20% m/v freshly activated 3 Å sieves for a minimum of 120 h.

### HfCl_4_·2THF and ZrCl_4_·2THF Precursor Synthesis

The procedure was slightly adapted from Manzer et al.^83^

#### HfCl_4_·2THF

22 g of HfCl_4_ (1 equiv, 0.069 mol) is added to 330 mL of anhydrous DCM, only partly dissolving. Next, 22 mL (3.95 equiv, 0.271 mol, 19.56 g) of anhydrous THF is added in a dropwise manner under vigorous stirring. The HfCl_4_·2THF dissolves completely while adding THF. 220 mL of dry pentane is carefully added along the sides, and the solution is placed in the freezer (−30 °C) for 2 h. The solution is filtered over a por 4 filter funnel. The resulting product is washed with 80 mL of dry pentane and dried overnight under vacuum, giving a white powder with yield up to 75%. The product is characterized by FTIR and ^1^H NMR (Figure S14A,B).

#### ZrCl_4_·2THF

11.65 g of ZrCl_4_ (1 equiv, 0.050 mol) is added to 150 mL of anhydrous DCM, only partly dissolving. Next, 8.11 mL (2 equiv, 0.100 mol, 7.21 g) of anhydrous THF is added in a dropwise manner under vigorous stirring. The ZrCl_4_·2THF dissolves while adding the THF; some turbidity remains. The solution is filtered over a por 4 filter to remove the insolubles. 125 mL of dry pentane is carefully added along the sides and the solution is placed in the freezer (−30 °C) for 2 h. The solution is filtered over a por 4 filter funnel. The resulting product is washed with 25 mL of dry pentane and dried overnight under vacuum, giving a white powder with yield up to 65%. The product is characterized by FTIR and ^1^H NMR (Figure S14C,D).

### Microwave-Assisted Solvothermal Synthesis of HfO_2_ Nanocrystals

The HfO_2_ nanocrystal synthesis is based on the original solvothermal synthesis by Buha et al.,^47^ which was adapted into a microwave-assisted synthesis by De Roo et al.^26^ and recently upscaled.^38^ The microwave procedure was conducted using a CEM Discover SP with an autosampler operating at a frequency of 2.45 GHz.

Synthesis preparation is executed in a nitrogen-filled glovebox. 0.372 g (1 equiv, 0.8 mmol) or 0.464 g (1 equiv, 1 mmol) of HfCl_4_·2THF is added to a 10 mL microwave vial with stirring bar. Under vigorous stirring, 4 mL (38 equiv or 48 equiv, 38 mmol, 4.16 g) of anhydrous benzyl alcohol is added to the microwave vial. The microwave vial is capped. The solution is then exited from the glovebox and stirred for 5 min, resulting in a transparent solution. The mixture is subjected to microwave heating for 5 min at 80 °C (30 W), followed by 4 h at 220 °C (300 W) at medium stirring and PowerMax off. After synthesis, the mixture is transferred to a 15 mL plastic centrifuge tube using a Pasteur pipet. The microwave vial is rinsed with 3 mL of diethyl ether in order to maximize the yield, after which this is also added to the centrifuge tube. After mild centrifugation (720 rcf, 2 min), three phases are observed: a transparent organic (top) phase, an aqueous, milky (middle) phase, and sometimes a solid (bottom) phase of insolubles. If the workup is done the same day as the synthesis, the solid phase is usually avoided. The transparent (top) phase is removed, and the milky phase is separated from the solid phase using a Pasteur pipet in a separate plastic centrifuge tube. The solid phase is discarded. Ethanol is added to the milky phase, yielding 2 mL of translucent suspension. Five mL of diethyl ether is added and the particles are precipitated (4500 rcf, 2 min), resulting in HfO_2_ nanocrystals capped with HCl.

#### Postsynthetic Yield Optimization

The synthesis is prepared as described above, but microwave heating is applied for 5 min at 80 °C (30 W), followed by only 1 h at 220 °C (300 W) at medium stirring and PowerMax off. After synthesis, 1 mL of distilled water and 20 μL of hydrochloric acid (37 w/w %) were added to the reaction mixture and subjected to sonication for 30 min. Afterward, the mixture is transferred to a 15 mL plastic centrifuge tube using a Pasteur pipet. The microwave vial is rinsed with 3 mL of diethyl ether, which is also added to the centrifuge tube. After mild centrifugation (720 rcf, 2 min), three phases are observed: a transparent organic (top) phase, an aqueous, milky (middle) phase, and sometimes a solid (bottom) phase of insolubles. The transparent (top) phase is removed, and the milky phase is separated from the solid phase using a Pasteur pipet in a separate plastic centrifuge tube. The solid phase is discarded. The milky phase is dried at the Schlenk line and redispersed in 2 mL ethanol. 5 mL of diethyl ether is added and the particles are precipitated (4500 rcf, 2 min), resulting in HfO_2_ nanocrystals capped with HCl.

#### Postsynthetic Surface Modification with Oleate

The particles are redispersed by sonication in 1 mL of chloroform, and 150 μL (0.47 mmol, 0.134 g) of oleic acid is added to the milky suspension. Next, 125 μL (0.38 mmol, 0.101 g) of oleylamine is added, instantly resulting in a transparent suspension. The particles are purified by adding 5 mL of acetone, followed by centrifigation (4500 rcf, 4 min), removal of the organic top phase, and resuspension in 1 mL of chloroform. This purification step was repeated three times. Sonication can be used to help redisperse the particles in the chloroform.

### Reaction Aliquots

In a nitrogen-filled glovebox, a 25 mL three-neck-flask, equipped with a small reflux condenser with vacuum adapter, thermowell, and silicon/PTFE septum, is loaded with 0.930 g (1 equiv, 2 mmol) of HfCl_4_·2THF and under vigorous stirring, 8 mL (38 equiv, 8.32 g) of anhydrous benzyl alcohol is added. The setup is exited from the glovebox, connected to the Schlenk line and flushed three times with Argon. Using the thermocontroller, the solution is first heated to 80 °C for 5 min and then to 200 °C while taking aliquots.

### Complexation with TOPO

In a nitrogen-filled glovebox, 11.6 mg (1 equiv, 0.025 mmol) HfCl_4_·2THF or 9.4 mg (1 equiv, 0.025 mmol) ZrCl_4_·2THF is dissolved in 0.5 mL C_6_D_6_ for each reaction, yielding a final concentration of 0.05 M. A 1 M stock solution of anhydrous benzyl alcohol in C_6_D_6_ was prepared. 0, 1, 2, or 4 equiv of benzyl alcohol was added using this 1 M stock solution. Each vial was evacuated and redispersed in 0.5 mL of C_6_D_6_ and 38.7 mg (4 equiv, 0.10 mmol) tri-*n*-octylphosphine oxide was added to the mixture. Samples needed to be evacuated to see the exchange. Figure S15A shows the NMR titration executed as described above but without the evacuation step. Samples were transferred to NMR tubes, and ^1^H and ^31^P NMR spectra were recorded.

### Yield Determination

Nanocrystals were synthesized, and the soluble NCs were functionalized with ligands, purified, and separated from the agglomerates as described above in “Microwave-Assisted Solvothermal Synthesis of HfO2 Nanocrystals”. For the optimized reaction, the protocol as described in “Postsynthetic Yield Optimization” was followed. Each sample was purified immediately after synthesis. Each data point was repeated three times. The final NCs were dried under vacuum and weighed. The ligand weight is subtracted from the mass. For each sample at 1 h of reaction time (three times the normal synthesis and three times the optimized synthesis) TGA data were measured to determine the exact mass loss. The other samples (2, 3, 4, and 5 h) of the first data set were measured with TGA as well. Since the TGA mass values did not change significantly between samples (standard deviation of <1%), the average ligand weight of all measurements was used for the second and third data set: 17.2 m %.

For each synthesis, the exact amount of precursor used is written down, which is used to calculate the maximum possible yield (in mg). The isolated yield is calculated by dividing the actual yield (ligand weigt subtracted) by the theoretical maximum possible yield. Table S4 shows the TGA mass loss, the sample weight, and the calculated yield of all data.

### Instruments and Characterization

#### TEM Analysis

High-resolution transmission electron microscopy (HRTEM) was performed on a JEOL JEM-2200FS TEM with Cs corrector and a JEOL JEM-F200 TEM, both operating at 200 kV. Samples were made by drop-cast suspensions on the grids. The TEM grids used were holey carbon-Cu (C200-CU) with 50 μm hole size (200 mesh). NC sizes were determined by measuring 200 particles using the “polygon selection” tool of ImageJ, with measurements set to “fit ellipse”.

#### NMR Analysis

Nuclear magnetic resonance (NMR) spectra were recorded at 298 K on a Bruker Avance III spectrometer operating at a ^1^H frequency of 500.13 MHz and featuring a BBI probe and a Bruker UltraShield 500 spectrometer operating at a ^1^H frequency of 500.13 MHz. Chemical shifts (δ) are given in parts per million (ppm), and the residual solvent peak was used as an internal standard (CDCl_3_: δH = 7.24 ppm and C_6_D_6_: δH = 7.16 ppm). For the quantitative 1D ^1^H measurements, 64k data points were sampled with the spectral width set to 16 ppm and a relaxation delay of 30s. Quantification was done using the Digital ERETIC method.^84^ Chemical shifts for ^31^P spectra were referenced indirectly to the ^1^H NMR frequency of the sample with the xiref-macro in Bruker.

#### PDF Analysis

*In situ* X-ray total scattering experiments were performed at the P02.1 PETRA III beamline at the DESY synchrotron, using a wavelength of λ = 0.207 34 Å. The RA-PDF geometry^85^ was applied with a large 2D detector (Varex XRD 4343CT) and a sample-to-detector distance of 263.0 mm. The synthesis was carried out in a custom-made reaction cell, similar to the design described by Becker et al.^86^ HfCl_4_·2THF was dissolved in BnOH, and the precursor suspension was injected into a fused silica tube with a 0.7 mm inner diameter and 0.09 mm wall thickness. The tube was pressurized by using a HPLC pump and heated by using a hot air blower. The collected 2D data were integrated using the Dioptas software,^87^ and the total scattering data were normalized and Fourier transformed using the PDFgetX3 software,^88^ to obtain the PDFs. *Q*_min_ = 1.2 Å^–1^, *Q*_max_ = 15.0 Å^–1^ and r_poly_ = 0.9 Å were used for data reduction. The background scattering signal from the fused silica capillary and pure benzyl alcohol was subtracted before the Fourier transformation. The PDFs were analysized with real-space Rietveld refinements using PDFgui.^89^ Monoclinic HfO_2_ crystal structure with space group *P*2_1_/*c* was used in the refinements.^61^ To follow the formation pathway, sequential refinement was performed. Nyquist data sampling was applied, and the refinements were initiated from the final crystalline product in the backward direction.

#### XRD Analysis

X-ray diffraction (XRD) was performed on a Bruker D8 Advance with motorized antiscatter screen and Autochanger and Bragg–Brentano θ–θ geometry (goniometer radius 280 mm). The instrument uses Cu Kα radiation (λ = 1.541 84 Å) with no Kβ filter. The detector is a LynxEye XE-T silicon strip line detector with 192 channels. Samples were made by drop-cast suspension on a glass plate. The measurement was performed in the 2θ 15–60° range at a step size of 0.02° and a scan rate of 0.5°/min.

#### XAS Analysis

X-ray absorption spectroscopy (XAS) analyses were performed at the SuperXAS beamline at PSI, monitoring the Zr–K (17.998 keV) and Hf-L_3_ (9.561 keV) edges. Data were processed using the Athena software package.^90^ Spectra were normalized to unity using the incident beam flux, *I*_0_. The energy axis was calibrated using standard reference compounds of Zr metallic foil and HfO_2_, respectively. Extended X-ray absorption fine structure (EXAFS) data were processed using the Demeter v0.9.26 software package,^90^ applying no low energy and strong high energy spline clamps. Data were analyzed using an amplitude reduction factor *S*_0_^2^ of 0.9, as derived from the reference spectra. Data were fit in k-space using the ranges 2–11.5 Å^–1^ and 1.5–14 Å^–1^ for the Hf- and Zr-species, respectively. Energy corrections to the experimental energy threshold value were applied uniformly to all paths simulated by a single FEFF calculation to ensure phase transferability between the experimental and theoretical EXAFS signals. Interatomic distances, thermal vibrations and scatter path degeneracy were allowed to fluctuate freely, in order to allow for as many degrees of freedom as possible.

#### FTIR Analysis

Fourier-transform infrared spectroscopy (FTIR) was performed on a PerkinElmer spectrum 2 ATR-FTIR with a diamond crystal measuring 8 scans from 450 to 4000 cm^–1^ and using background subtraction.

#### Rheology Measurements

Rheology measurements were performed by using an Anton-Paar MCR 302 rheometer with a parallel plate geometry. The storage and loss moduli are measured at a certain rotational speed by heating 0.25 M HfCl_4_·2THF or ZrCl_4_·2THF in benzyl alcohol from 25 °C to 220 °C at a rate of 3 °C/min while measuring a data point every 130 s using a shear strain of 1% and a frequency of 1 Hz. A solvent trap of benzyl alcohol was used to minimize the level of evaporation. To ensure that measurements were performed in the linear viscoelastic region, both a frequency and amplitude sweep was performed after heating 0.25 M HfCl_4_·2THF in benzyl alcohol to 160 °C. For the recovery experiments, the gel was created using microwave heating and quenched to room temperature. The mixture was either measured at room temperature or re-equilibrated at 160 °C, after which a 10% shear stress was applied at 1 Hz for 10 s to disturb the system, followed by monitoring of the recovery over a period of 60 s at 1% shear stress. This cycle was repeated 3 times total.

## Supporting Information Available

Additional rheology, EXAFS, NMR, and PDF data, DFT precursor structures, control experiments, tables of EXAFS and PDF fitting, and NMR results (PDF)
Microwave video (MP4)
xyz files of DFT calculated precursor structures (ZIP)

## References

[ref1] Danks A. E.; Hall S. R.; Schnepp Z. The evolution of ‘sol–gel’chemistry as a technique for materials synthesis. Materials Horizons 2016, 3, 91–112. 10.1039/C5MH00260E.

[ref2] Brinker C. J.; Scherer G. W. Sol-Gel Science: The Physics and Chemistry of Sol-Gel Processing; Academic Press, 2013.

[ref3] Livage J.; Sanchez C. Sol-gel chemistry. J. Non-Cryst. Solids 1992, 145, 11–19. 10.1016/S0022-3093(05)80422-3.

[ref4] Hench L. L.; West J. K. The sol-gel process. Chem. Rev. 1990, 90, 33–72. 10.1021/cr00099a003.

[ref5] Narendar Y.; Messing G. L. Mechanisms of phase separation in gel-based synthesis of multicomponent metal oxides. Catal. Today 1997, 35, 247–268. 10.1016/S0920-5861(96)00160-5.

[ref6] Pinna N.; Karmaoui M.; Willinger M.-G. The “benzyl alcohol route”: An elegant approach towards doped and multimetal oxide nanocrystals: Short review and ZnAl 2 O 4 nanostructures by oriented attachment. J. Sol-Gel Sci. Technol. 2011, 57, 323–329. 10.1007/s10971-009-2111-2.

[ref7] Andrianainarivelo M.; Corriu R. J.; Leclercq D.; Mutin P. H.; Vioux A. Nonhydrolytic Sol-Gel process: Aluminium and zirconium titanate gels. J. Sol-Gel Sci. Technol. 1997, 8, 89–93. 10.1007/BF02436823.

[ref8] Corriu R. J.; Leclercq D.; Lefèvre P.; Mutin P. H.; Vioux A. Materials chemistry communications. Preparation of monolithic metal oxide gels by a non-hydrolytic sol–gel process. J. Mater. Chem. 1992, 2, 673–674. 10.1039/JM9920200673.

[ref9] Arnal P.; Corriu R. J.; Leclercq D.; Mutin P. H.; Vioux A. Preparation of anatase, brookite and rutile at low temperature by non-hydrolytic sol–gel methods. J. Mater. Chem. 1996, 6, 1925–1932. 10.1039/JM9960601925.

[ref10] Arnal P.; Corriu R. J.; Leclercq D.; Mutin P. H.; Vioux A. Preparation of transition metal oxides by a nonhydrolytic sol-gel process. MRS Online Proc. Libr. 1994, 346, 339–344. 10.1557/PROC-346-339.

[ref11] Arnal P.; Corriu R. J.; Leclercq D.; Mutin P. H.; Vioux A. A Solution Chemistry Study of Nonhydrolytic Sol- Gel Routes to Titania. Chemistry of materials 1997, 9, 694–698. 10.1021/cm960337t.

[ref12] Niederberger M.; Garnweitner G. Organic reaction pathways in the nonaqueous synthesis of metal oxide nanoparticles. Chem.—Eur. J. 2006, 12, 7282–7302. 10.1002/chem.200600313. 16927442

[ref13] Niederberger M.; Bartl M. H.; Stucky G. D. Benzyl alcohol and transition metal chlorides as a versatile reaction system for the nonaqueous and low-temperature synthesis of crystalline nano-objects with controlled dimensionality. J. Am. Chem. Soc. 2002, 124, 13642–13643. 10.1021/ja027115i. 12431071

[ref14] Niederberger M. Nonaqueous sol–gel routes to metal oxide nanoparticles. Accounts of chemical research 2007, 40, 793–800. 10.1021/ar600035e. 17461544

[ref15] Niederberger M.; Pinna N. Aqueous and nonaqueous sol-gel chemistry. Metal Oxide Nanoparticles in Organic Solvents: Synthesis, Formation, Assembly and Application; Springer: London, 2009; pp 7–18, 10.1007/978-1-84882-671-7_2.

[ref16] Heiligtag F. J.; Niederberger M. The fascinating world of nanoparticle research. Mater. Today 2013, 16, 262–271. 10.1016/j.mattod.2013.07.004.

[ref17] Deshmukh R.; Niederberger M. Mechanistic Aspects in the Formation, Growth and Surface Functionalization of Metal Oxide Nanoparticles in Organic Solvents. Chem.—Eur. J. 2017, 23, 8542–8570. 10.1002/chem.201605957. 28376243

[ref18] Van den Eynden D.; Pokratath R.; De Roo J. Nonaqueous Chemistry of Group 4 Oxo Clusters and Colloidal Metal Oxide Nanocrystals. Chem. Rev. 2022, 122, 10538–10572. 10.1021/acs.chemrev.1c01008. 35467844

[ref19] Jennings J.; Wardlaw W.; Way W. 146. Some esters of titanium. Journal of the Chemical Society (Resumed) 1936, 637–640. 10.1039/jr9360000637.

[ref20] Bradley D.; Abd-el Halim F.; Wardlaw W. 676. The chloride ethoxides of zirconium. Journal of the Chemical Society (Resumed) 1950, 3450–3454. 10.1039/jr9500003450.

[ref21] Simmons C. R.; Hansen R. S. Solvolysis of Hafnium and Zirconium Tetrachlorides in Methyl and Ethyl Alcohols. J. Phys. Chem. 1955, 59, 1072–1073. 10.1021/j150532a020.

[ref22] Bradley D.; Saad M.; Wardlaw W. The preparation of thorium alkoxides. Journal of the Chemical Society (Resumed) 1954, 1091–1094. 10.1039/jr9540001091.

[ref23] Kløve M.; Christensen R. S.; Nielsen I. G.; Sommer S.; Jørgensen M. R. V.; Dippel A.-C.; Iversen B. B. Zr 4+ solution structures from pair distribution function analysis. Chemical Science 2022, 13, 12883–12891. 10.1039/D2SC04522B. 36519061 PMC9645415

[ref24] Jensen G. V.; Bremholm M.; Lock N.; Deen G. R.; Jensen T. R.; Iversen B. B.; Niederberger M.; Pedersen J. S.; Birkedal H. Anisotropic crystal growth kinetics of anatase TiO2 nanoparticles synthesized in a nonaqueous medium. Chem. Mater. 2010, 22, 6044–6055. 10.1021/cm100469y.

[ref25] Zimmermann M.; Garnweitner G. Spontaneous water release inducing nucleation during the nonaqueous synthesis of TiO 2 nanoparticles. CrystEngComm 2012, 14, 8562–8568. 10.1039/c2ce25934f.

[ref26] De Roo J.; De Keukeleere K.; Feys J.; Lommens P.; Hens Z.; Van Driessche I. Fast, microwave-assisted synthesis of monodisperse HfO2 nanoparticles. J. Nanopart. Res. 2013, 15, 1778 10.1007/s11051-013-1778-z.

[ref27] Aalling-Frederiksen O.; Juelsholt M.; Anker A. S.; Jensen K. M. Formation and growth mechanism for niobium oxide nanoparticles: atomistic insight from in situ X-ray total scattering. Nanoscale 2021, 13, 8087–8097. 10.1039/D0NR08299F. 33956920 PMC8101635

[ref28] Gambe J.; Remondiere F.; Jouin J.; Portal L.; Thomas P.; Masson O. Detrimental Effect and Neutralization of in Situ Produced Water on Zirconia Nanoparticles Obtained by a Nonaqueous Sol–Gel Method. Inorganic chemistry 2019, 58, 15175–15188. 10.1021/acs.inorgchem.9b02076. 31663336

[ref29] Xie S.; Iglesia E.; Bell A. T. Water-assisted tetragonal-to-monoclinic phase transformation of ZrO2 at low temperatures. Chemistry of materials 2000, 12, 2442–2447. 10.1021/cm000212v.

[ref30] Auxéméry A.; Philippot G.; Suchomel M. R.; Testemale D.; Aymonier C. Stabilization of tetragonal zirconia nanocrystallites using an original supercritical-based synthesis route. Chem. Mater. 2020, 32, 8169–8181. 10.1021/acs.chemmater.0c01550.

[ref31] De Keukeleere K.; De Roo J.; Lommens P.; Martins J. C.; Van Der Voort P.; Van Driessche I. Fast and tunable synthesis of ZrO2 nanocrystals: mechanistic insights into precursor dependence. Inorganic chemistry 2015, 54, 3469–3476. 10.1021/acs.inorgchem.5b00046. 25751155

[ref32] Lauria A.; Villa I.; Fasoli M.; Niederberger M.; Vedda A. Multifunctional role of rare earth doping in optical materials: Nonaqueous sol–gel synthesis of stabilized cubic HfO2 luminescent nanoparticles. ACS Nano 2013, 7, 7041–7052. 10.1021/nn402357s. 23898781

[ref33] Rauwel P.; Galeckas A.; Rauwel E. One Step Synthesis of Pure Cubic and Monoclinic HfO2 Nanoparticles: Effects of Temperature and Ambient on the Photoluminescent Properties. ECS Trans. 2015, 64, 19 10.1149/06444.0019ecst.

[ref34] Wang J.; Choudhary S.; De Roo J.; De Keukeleere K.; Van Driessche I.; Crosby A. J.; Nonnenmann S. S. How ligands affect resistive switching in solution-processed HfO2 nanoparticle assemblies. ACS Appl. Mater. Interfaces 2018, 10, 4824–4830. 10.1021/acsami.7b17376. 29338165

[ref35] De Roo J.; Zhou Z.; Wang J.; Deblock L.; Crosby A. J.; Owen J. S.; Nonnenmann S. S. Synthesis of phosphonic acid ligands for nanocrystal surface functionalization and solution processed memristors. Chem. Mater. 2018, 30, 8034–8039. 10.1021/acs.chemmater.8b03768.

[ref36] Maiti S.; Ohlerth T.; Schmidt N.; Aussen S.; Waser R.; Simon U.; Karthäuser S. Moisture Effect on the Threshold Switching of TOPO-Stabilized Sub-10 nm HfO2 Nanocrystals in Nanoscale Devices. J. Phys. Chem. C 2022, 126, 18571–18579. 10.1021/acs.jpcc.2c06303.

[ref37] McGinnity T. L.; Dominguez O.; Curtis T. E.; Nallathamby P. D.; Hoffman A. J.; Roeder R. K. Hafnia (HfO 2) nanoparticles as an X-ray contrast agent and mid-infrared biosensor. Nanoscale 2016, 8, 13627–13637. 10.1039/C6NR03217F. 27364973

[ref38] Goossens E.; et al. From corrosion casting to virtual dissection: contrast-enhanced vascular imaging using hafnium oxide nanocrystals. Small Methods 2024, 2301499 10.1002/smtd.202301499. 38200600

[ref39] Deblock L.; Descamps B.; Goemaere I.; Goossens E.; Vergauwen G.; Debacker J.; Tummers P.; Remaut K.; Van Driessche I.; De Buysser K.; De Roo J.; Vanhove C. Dual-Modality Hafnium Oxide Nanocrystals for in Vivo Computed Tomography and Fluorescence Imaging of Sentinel Lymph Nodes. Chem. Mater. 2023, 35, 8883–8896. 10.1021/acs.chemmater.3c01324.

[ref40] Sebti Y.; Si-Mohamed S.; Aid R.; Geinguenaud F.; Chalal M.; Lalatonne Y.; Chaubet F.; Ou P.; Motte L. Optical and X-ray attenuation properties of hafnium oxide nanoparticles surface functionalized with fucoidan: toward the early diagnosis of atherothrombotic diseases. Materials Advances 2023, 4, 1011–1020. 10.1039/D2MA01026G.

[ref41] Ostadhossein F.; Tripathi I.; Benig L.; LoBato D.; Moghiseh M.; Lowe C.; Raja A.; Butler A.; Panta R.; Anjomrouz M.; Chernoglazov A.; Pan D. Multi-“Color” Delineation of Bone Microdamages Using Ligand-Directed Sub-5 nm Hafnia Nanodots and Photon Counting CT Imaging. Adv. Funct. Mater. 2020, 30, 1904936 10.1002/adfm.201904936.

[ref42] Ostadhossein F.; Moitra P.; Gunaseelan N.; Nelappana M.; Lowe C.; Moghiseh M.; Butler A.; de Ruiter N.; Mandalika H.; Tripathi I.; et al. Hitchhiking probiotic vectors to deliver ultra-small hafnia nanoparticles for ‘Color’gastrointestinal tract photon counting X-ray imaging. Nanoscale Horizons 2022, 7, 533–542. 10.1039/D1NH00626F. 35311837

[ref43] Liu C.; Hajagos T. J.; Kishpaugh D.; Jin Y.; Hu W.; Chen Q.; Pei Q. Facile single-precursor synthesis and surface modification of hafnium oxide nanoparticles for nanocomposite γ-ray scintillators. Adv. Funct. Mater. 2015, 25, 4607–4616. 10.1002/adfm.201501439.

[ref44] Yu H.; Winardi I.; Han Z.; Prout D.; Chatziioannou A.; Pei Q. Fast Spectroscopic Gamma Scintillation Using Hafnium Oxide Nanoparticles–Plastic Nanocomposites. Chem. Mater. 2024, 36, 533–540. 10.1021/acs.chemmater.3c02631.

[ref45] De Roo J.; Van Driessche I.; Martins J. C.; Hens Z. Colloidal metal oxide nanocrystal catalysis by sustained chemically driven ligand displacement. Nature materials 2016, 15, 517–521. 10.1038/nmat4554. 26808460

[ref46] Maggiorella L.; Barouch G.; Devaux C.; Pottier A.; Deutsch E.; Bourhis J.; Borghi E.; Levy L. Nanoscale radiotherapy with hafnium oxide nanoparticles. Future oncology 2012, 8, 1167–1181. 10.2217/fon.12.96. 23030491

[ref47] Buha J.; Arčon D.; Niederberger M.; Djerdj I. Solvothermal and surfactant-free synthesis of crystalline Nb 2 O 5, Ta 2 O 5, HfO 2, and Co-doped HfO 2 nanoparticles. Phys. Chem. Chem. Phys. 2010, 12, 15537–15543. 10.1039/c0cp01298j. 20976360

[ref48] De Roo J.; Van den Broeck F.; De Keukeleere K.; Martins J. C.; Van Driessche I.; Hens Z. Unravelling the surface chemistry of metal oxide nanocrystals, the role of acids and bases. J. Am. Chem. Soc. 2014, 136, 9650–9657. 10.1021/ja5032979. 24945901

[ref49] Deblock L.; Goossens E.; Pokratath R.; De Buysser K.; De Roo J. Mapping out the Aqueous Surface Chemistry of Metal Oxide Nanocrystals: Carboxylate, Phosphonate, and Catecholate Ligands. JACS Au 2022, 2, 711–722. 10.1021/jacsau.1c00565. 35373200 PMC8969999

[ref50] Pokratath R.; Van den Eynden D.; Cooper S. R.; Mathiesen J. K.; Waser V.; Devereux M.; Billinge S. J.; Meuwly M.; Jensen K. M.; De Roo J. Mechanistic Insight into the Precursor Chemistry of ZrO2 and HfO2 Nanocrystals; towards Size-Tunable Syntheses. JACS Au 2022, 2, 827–838. 10.1021/jacsau.1c00568. 35557760 PMC9088301

[ref51] Duraj S.; Towns R.; Baker R.; Schupp J. Structure of cis-tetrachlorobis (tetrahydrofuran) hafnium (IV). Acta Crystallographica Section C: Crystal Structure Communications 1990, 46, 890–892. 10.1107/S010827018901382X.

[ref52] Clayden J.; Greeves N.; Warren S. Organic Chemistry; Oxford University Press: USA, 2012.

[ref53] Sakka S. Formation of glass and amorphous oxide fibers from solution. MRS Online Proc. Libr. 1984, 32, 91–99. 10.1557/PROC-32-91.

[ref54] Mizuno T.; Phalippou J.; Zarzycki J. Evolution of the viscosity of solutions containing metal alkoxides. Glass Technol. 1985, 26, 39–45.

[ref55] Kesslin G.; Bradshaw R. Ortho Esters as Water Scavengers. Industrial & Engineering Chemistry Product Research and Development 1966, 5, 27–29. 10.1021/i360017a005.

[ref56] Juelsholt M.; Lindahl Christiansen T.; Jensen K. M. Mechanisms for tungsten oxide nanoparticle formation in solvothermal synthesis: from polyoxometalates to crystalline materials. J. Phys. Chem. C 2019, 123, 5110–5119. 10.1021/acs.jpcc.8b12395.

[ref57] Jensen K. M.; Christensen M.; Juhas P.; Tyrsted C.; Bøjesen E. D.; Lock N.; Billinge S. J.; Iversen B. B. Revealing the mechanisms behind SnO2 nanoparticle formation and growth during hydrothermal synthesis: an in situ total scattering study. J. Am. Chem. Soc. 2012, 134, 6785–6792. 10.1021/ja300978f. 22420861

[ref58] Jensen K. M.; Andersen H. L.; Tyrsted C.; Bøjesen E. D.; Dippel A.-C.; Lock N.; Billinge S. J.; Iversen B. B.; Christensen M. Mechanisms for iron oxide formation under hydrothermal conditions: an in situ total scattering study. ACS Nano 2014, 8, 10704–10714. 10.1021/nn5044096. 25256366

[ref59] Bøjesen E. D.; Jensen K. M.; Tyrsted C.; Mamakhel A.; Andersen H. L.; Reardon H.; Chevalier J.; Dippel A.-C.; Iversen B. B. The chemistry of ZnWO 4 nanoparticle formation. Chemical Science 2016, 7, 6394–6406. 10.1039/C6SC01580H. 28451095 PMC5355961

[ref60] Christensen R. S.; Kløve M.; Roelsgaard M.; Sommer S.; Iversen B. B. Unravelling the complex formation mechanism of HfO 2 nanocrystals using in situ pair distribution function analysis. Nanoscale 2021, 13, 12711–12719. 10.1039/D1NR03044B. 34477621

[ref61] Pathak S.; Das P.; Das T.; Mandal G.; Joseph B.; Sahu M.; Kaushik S.; Siruguri V. Crystal structure of monoclinic hafnia (HfO2) revisited with synchrotron X-ray, neutron diffraction and first-principles calculations. Acta Crystallographica Section C: Structural Chemistry 2020, 76, 1034–1042. 10.1107/S2053229620013960. 33148879

[ref62] Tyrsted C.; Lock N.; Jensen K.; Christensen M.; Bøjesen E. D.; Emerich H.; Vaughan G.; Billinge S. J.; Iversen B. B. Evolution of atomic structure during nanoparticle formation. IUCrJ. 2014, 1, 165–171. 10.1107/S2052252514006538. 25075335 PMC4086431

[ref63] Song Q.; Zhang Z. J. Shape control and associated magnetic properties of spinel cobalt ferrite nanocrystals. J. Am. Chem. Soc. 2004, 126, 6164–6168. 10.1021/ja049931r. 15137781

[ref64] Joo J.; Kwon S. G.; Yu J. H.; Hyeon T. Synthesis of ZnO nanocrystals with cone, hexagonal cone, and rod shapes via non-hydrolytic ester elimination sol–gel reactions. Adv. Mater. 2005, 17, 1873–1877. 10.1002/adma.200402109.

[ref65] Li X.-L.; Peng Q.; Yi J.-X.; Wang X.; Li Y. Near monodisperse TiO2 nanoparticles and nanorods. Chem.—Eur. J. 2006, 12, 2383–2391. 10.1002/chem.200500893. 16374889

[ref66] Polleux J.; Gurlo A.; Barsan N.; Weimar U.; Antonietti M.; Niederberger M. Template-free synthesis and assembly of single-crystalline tungsten oxide nanowires and their gas-sensing properties. Angew. Chem., Int. Ed. 2006, 45, 261–265. 10.1002/anie.200502823. 16312002

[ref67] Abulikemu M.; Tietze M. L.; Waiprasoet S.; Pattanasattayavong P.; Tabrizi B. E. A.; D’Elia V.; Del Gobbo S.; Jabbour G. E. Microwave-Assisted Non-aqueous and Low-Temperature Synthesis of Titania and Niobium-Doped Titania Nanocrystals and Their Application in Halide Perovskite Solar Cells as Electron Transport Layers. ACS Omega 2022, 7, 6616–6626. 10.1021/acsomega.1c05970. 35252657 PMC8892854

[ref68] Štefanić G.; Musić S.; Molčanov K. The crystallization process of HfO2 and ZrO2 under hydrothermal conditions. Journal of alloys and compounds 2005, 387, 300–307. 10.1016/j.jallcom.2004.06.064.

[ref69] Eliziario S.; Cavalcante L.; Sczancoski J.; Pizani P.; Varela J. A.; Espinosa J.; Longo E. Morphology and photoluminescence of HfO2 obtained by microwave-hydrothermal. Nanoscale Res. Lett. 2009, 4, 1371 10.1007/s11671-009-9407-6. 20628455 PMC2893942

[ref70] Sahraneshin A.; Asahina S.; Togashi T.; Singh V.; Takami S.; Hojo D.; Arita T.; Minami K.; Adschiri T. Surfactant-assisted hydrothermal synthesis of water-dispersible hafnium oxide nanoparticles in highly alkaline media. Cryst. Growth Des. 2012, 12, 5219–5226. 10.1021/cg3005739.

[ref71] Wan Y.; Zhou X. Formation mechanism of hafnium oxide nanoparticles by a hydrothermal route. RSC Adv. 2017, 7, 7763–7773. 10.1039/C6RA26663K.

[ref72] De Roo J. Chemical Considerations for Colloidal Nanocrystal Synthesis. Chem. Mater. 2022, 34, 5766–5779. 10.1021/acs.chemmater.2c01058.

[ref73] LaMer V. K.; Dinegar R. H. Theory, production and mechanism of formation of monodispersed hydrosols. Journal of the American Chemical Society 1950, 72, 4847–4854. 10.1021/ja01167a001.

[ref74] Sugimoto T. Underlying mechanisms in size control of uniform nanoparticles. J. Colloid Interface Sci. 2007, 309, 106–118. 10.1016/j.jcis.2007.01.036. 17336993

[ref75] Pokratath R.; Lermusiaux L.; Checchia S.; Mathew J. P.; Cooper S. R.; Mathiesen J. K.; Landaburu G.; Banerjee S.; Tao S.; Reichholf N.; et al. An Amorphous Phase Precedes Crystallization: Unraveling the Colloidal Synthesis of Zirconium Oxide Nanocrystals. ACS Nano 2023, 17, 8796–8806. 10.1021/acsnano.3c02149. 37093055 PMC10173684

[ref76] Habraken W. J.; Tao J.; Brylka L. J.; Friedrich H.; Bertinetti L.; Schenk A. S.; Verch A.; Dmitrovic V.; Bomans P. H.; Frederik P. M.; et al. Ion-association complexes unite classical and non-classical theories for the biomimetic nucleation of calcium phosphate. Nat. Commun. 2013, 4, 1507 10.1038/ncomms2490. 23422675

[ref77] Baumgartner J.; Dey A.; Bomans P. H.; Le Coadou C.; Fratzl P.; Sommerdijk N. A.; Faivre D. Nucleation and growth of magnetite from solution. Nature materials 2013, 12, 310–314. 10.1038/nmat3558. 23377292

[ref78] Yang J.; Koo J.; Kim S.; Jeon S.; Choi B. K.; Kwon S.; Kim J.; Kim B. H.; Lee W. C.; Lee W. B.; et al. Amorphous-phase-mediated crystallization of Ni nanocrystals revealed by high-resolution liquid-phase electron microscopy. J. Am. Chem. Soc. 2019, 141, 763–768. 10.1021/jacs.8b11972. 30608684

[ref79] Montanarella F.; Akkerman Q. A.; Bonatz D.; van der Sluijs M. M.; van der Bok J. C.; Prins P. T.; Aebli M.; Mews A.; Vanmaekelbergh D.; Kovalenko M. V. Growth and Self-Assembly of CsPbBr3 Nanocrystals in the TOPO/PbBr2 Synthesis as Seen with X-ray Scattering. Nano Lett. 2023, 23, 667–676. 10.1021/acs.nanolett.2c04532. 36607192 PMC9881167

[ref80] Livage J.; Henry M.; Sanchez C. Sol-gel chemistry of transition metal oxides. Progress in solid state chemistry 1988, 18, 259–341. 10.1016/0079-6786(88)90005-2.

[ref81] Owen J. S.; Park J.; Trudeau P.-E.; Alivisatos A. P. Reaction chemistry and ligand exchange at cadmium- selenide nanocrystal surfaces. J. Am. Chem. Soc. 2008, 130, 12279–12281. 10.1021/ja804414f. 18722426

[ref82] Williams D. B. G.; Lawton M. Drying of organic solvents: quantitative evaluation of the efficiency of several desiccants. Journal of organic chemistry 2010, 75, 8351–8354. 10.1021/jo101589h. 20945830

[ref83] Manxzer L.; Deaton J.; Sharp P.; Schrock R. 31. Tetragtdrfuran Complexes of Selected Early Transition Metals. Inorganic Syntheses 1982, 21, 135–140. 10.1002/9780470132524.ch31.

[ref84] Akoka S.; Barantin L.; Trierweiler M. Concentration measurement by proton NMR using the ERETIC method. Anal. Chem. 1999, 71, 2554–2557. 10.1021/ac981422i. 21662801

[ref85] Chupas P. J.; Qiu X.; Hanson J. C.; Lee P. L.; Grey C. P.; Billinge S. J. Rapid-acquisition pair distribution function (RA-PDF) analysis. J. Appl. Crystallogr. 2003, 36, 1342–1347. 10.1107/S0021889803017564.

[ref86] Becker J.; Bremholm M.; Tyrsted C.; Pauw B.; Jensen K.; Eltzholt J.; Christensen M.; Iversen B. B. Experimental setup for in situ X-ray SAXS/WAXS/PDF studies of the formation and growth of nanoparticles in near-and supercritical fluids. J. Appl. Crystallogr. 2010, 43, 729–736. 10.1107/S0021889810014688.

[ref87] Prescher C.; Prakapenka V. B. DIOPTAS: a program for reduction of two-dimensional X-ray diffraction data and data exploration. High Pressure Research 2015, 35, 223–230. 10.1080/08957959.2015.1059835.

[ref88] Juhás P.; Davis T.; Farrow C. L.; Billinge S. J. PDFgetX3: a rapid and highly automatable program for processing powder diffraction data into total scattering pair distribution functions. J. Appl. Crystallogr. 2013, 46, 560–566. 10.1107/S0021889813005190.

[ref89] Farrow C.; Juhas P.; Liu J.; Bryndin D.; Božin E.; Bloch J.; Proffen T.; Billinge S. PDFfit2 and PDFgui: computer programs for studying nanostructure in crystals. J. Phys.: Condens. Matter 2007, 19, 335219 10.1088/0953-8984/19/33/335219. 21694142

[ref90] Ravel B.; Newville M. ATHENA, ARTEMIS, HEPHAESTUS: data analysis for X-ray absorption spectroscopy using IFEFFIT. J. Synchrotron Radiat 2005, 12, 537–41. 10.1107/S0909049505012719. 15968136

