# Supplementary material for: From Gel to Crystal: Mechanism of HfO2 and ZrO2 Nanocrystal Synthesis in Benzyl Alcohol
Source: J Am Chem Soc. 2024 Apr 8;146(15):10723–34. doi: 10.1021/jacs.4c00678 (PMC11027147; doi:10.1021/jacs.4c00678)
Supplement: Supplementary file 1 — ja4c00678_si_001.pdf [file ja4c00678_si_001.pdf]

Supporting Information: From gel to crystal: mechanism of HfO_2 and ZrO_2 nanocrystal synthesis in benzyl alcohol

Eline Goossens,[†,‡] Olivia Aalling-Frederiksen,[¶] Pieter Tack,[†] Dietger Van den Eynden,[‡,†] Zarah Walsh–Korb,[‡] Kirsten M Ø Jensen,[¶] Klaartje De Buysser,[†] and Jonathan De Roo[*,‡]

†*Department of Chemistry, Ghent University, 9000 Ghent, Belgium*

‡*Department of Chemistry, University of Basel, 4058 Basel, Switzerland*

¶*Department of Chemistry, University of Copenhagen, Copenhagen 2100, Denmark*

E-mail: Jonathan.DeRoo@unibas.ch

Main figures

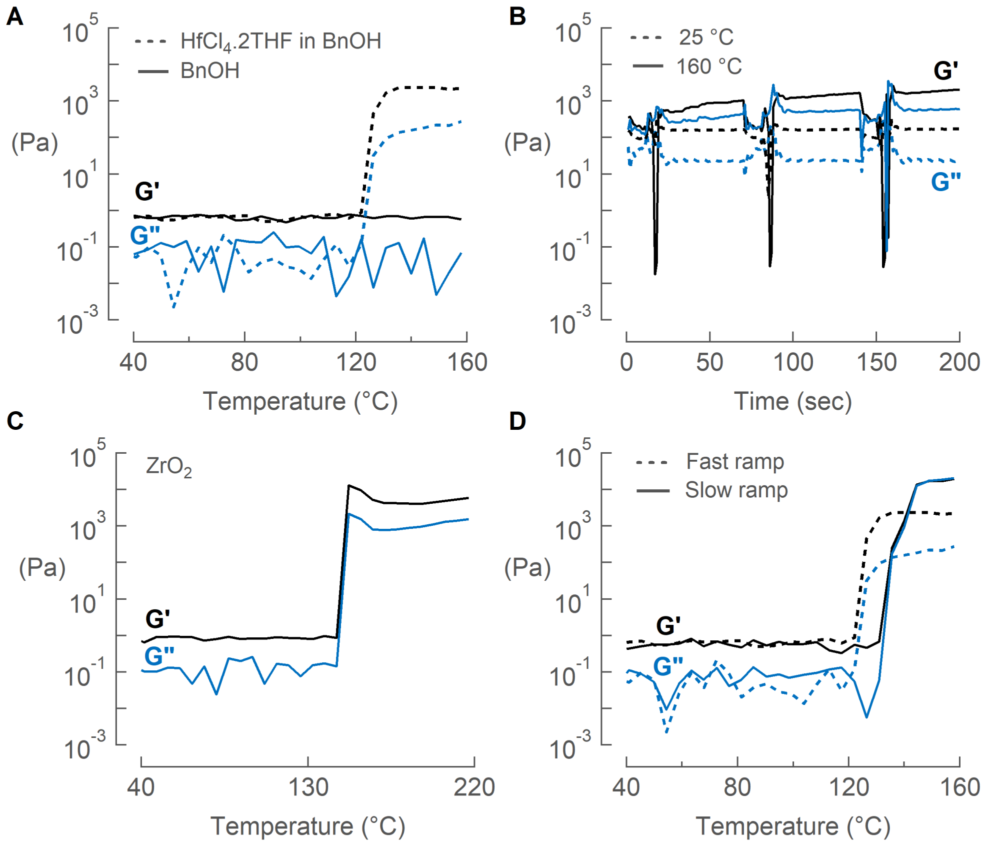

Figure S1: (A) Storage modulus G' and loss modulus G" with increasing temperature of benzyl alcohol in overlay with the measurement for 0.25M $HfCl_4$.2THF in benzyl alcohol. (B) Recovery cycles of G' and G" after applying stress to the gel, both at 25 °C and 160 °C. G' and G" with increasing temperature of (C) 0.25M $ZrCl_4$.2THF in benzyl alcohol, a sharp viscosity increase between 140 °C and 150 °C is noted, and (C) 0.25M $HfCl_4$.2THF in benzyl alcohol with a fast (0.03 °C/sec) and slow (0.01 °C/sec) heating ramp.

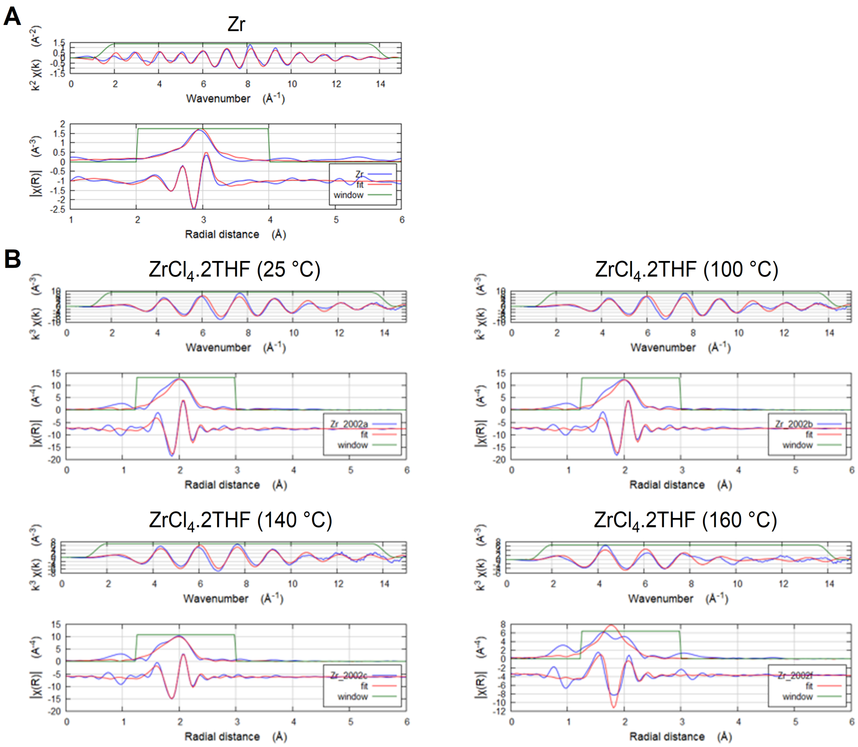

Figure S2: EXAFS k-vs-fit curves of Zr-O and Zr-Cl single scatter path (R-space).

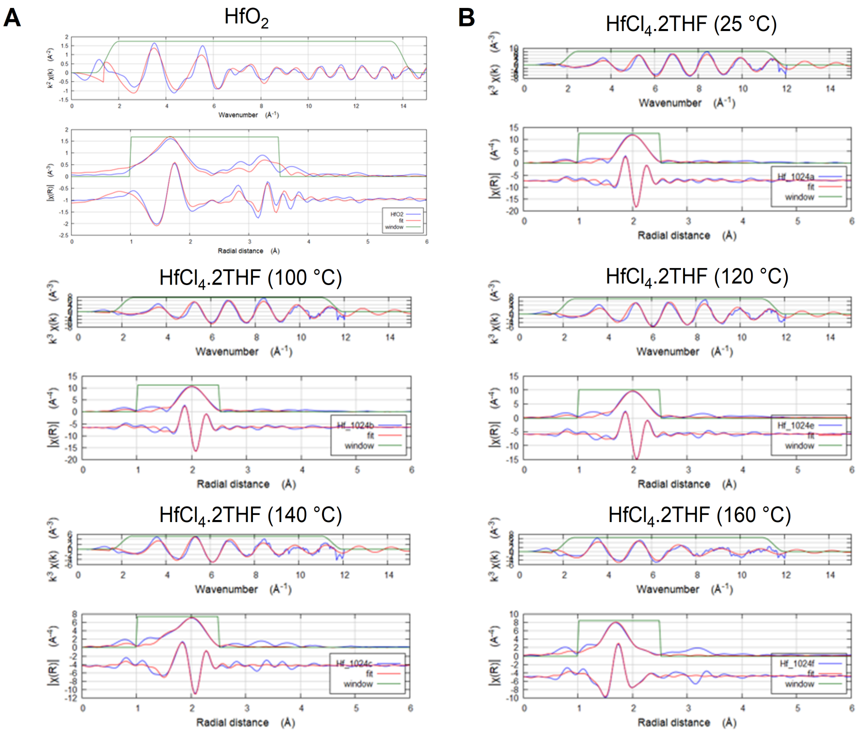

Figure S3: EXAFS k-vs-fit curves of Hf-O and Hf-Cl single scatter path (k-space).

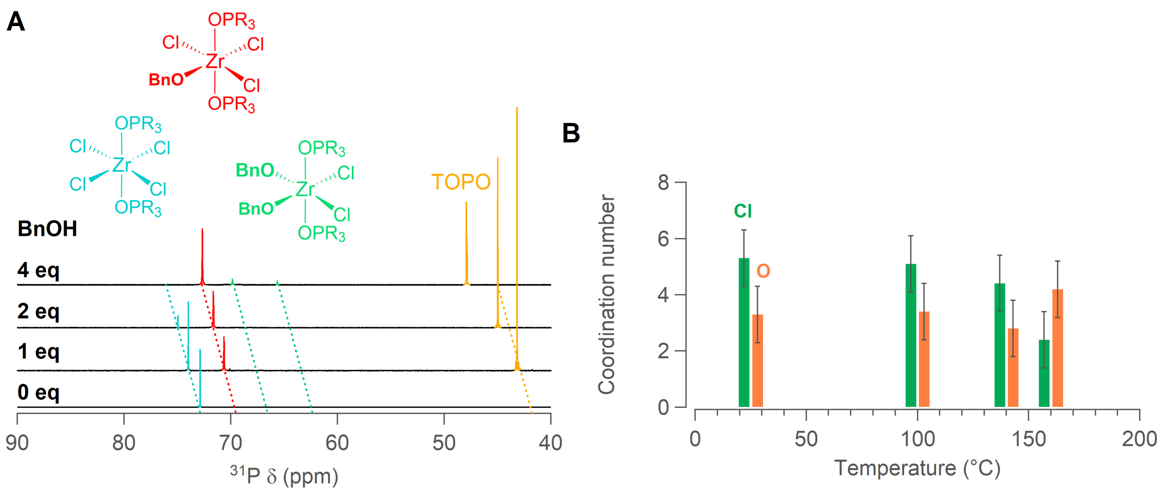

Figure S4: (A) Titration of $ZrCl_4.2THF$ with benzyl alcohol in C_6D_6, followed via ^{31}P NMR. The spectra have a relative x-offset of 1 ppm with respect to each other for clarity. (B) Coordination numbers of chloride and oxygen surrounding the zirconium center, calculated from the EXAFS data.

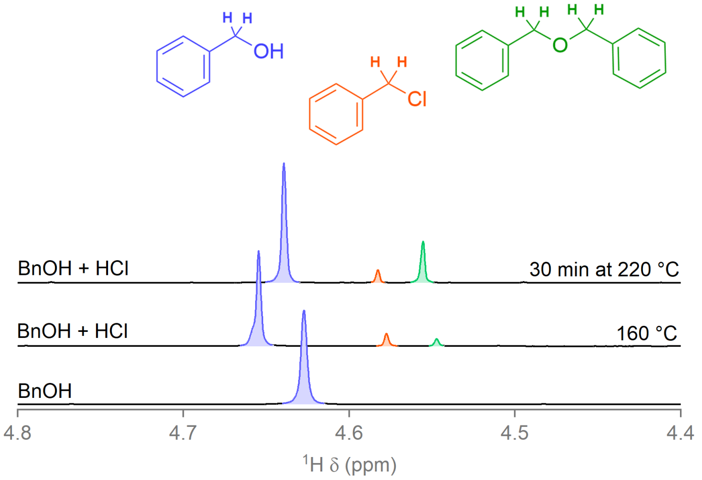

Figure S5: A control experiment of 38 mmol benzyl alcohol with 2.5 mmol HCl (37 w/w%) shows that both benzyl chloride and dibenzyl ether are present at 160 °C and at 220 °C and therefore are created by the reaction between benzyl alcohol and HCl. No gel phase is observed with the microwave camera.

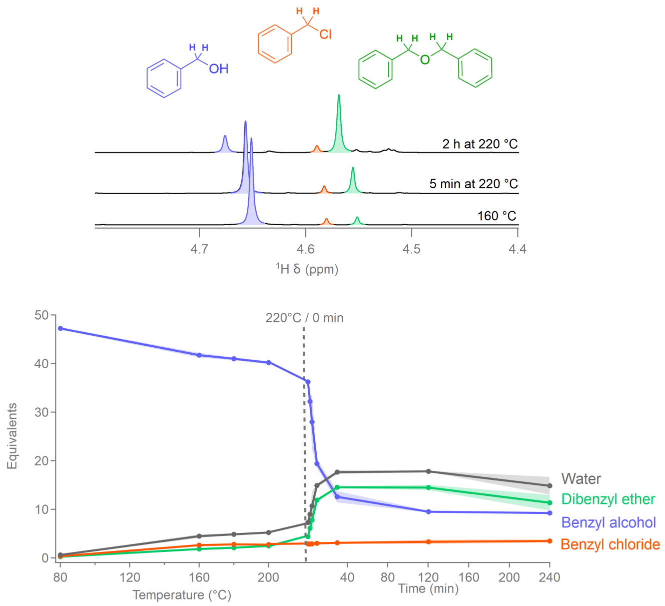

Figure S6: Assignment of benzyl alcohol, benzyl chloride and dibenzyl ether in the region between 4.4 and 4.8 ppm for ^{1}H NMR (top). The equivalents calculated from ^{1}H NMR spectra for a solution of 0.20M $HfCl_4$.2THF dissolved in benzyl alcohol where the microwave reaction is stopped at different temperature and time points. Each synthesis was done separately and in triplicate, shading indicates the error bars. See table S1 for exact values in %. The water content is calculated based on the conversion of benzyl alcohol to dibenzyl ether and benzyl chloride.

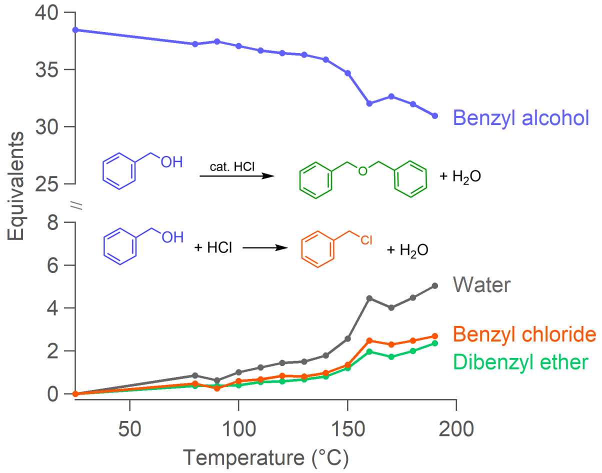

Figure S7: Equivalents of benzyl alcohol, water, benzyl chloride and dibenzyl ether in the reaction mixture of 0.25M $ZrCl_4$.2THF dissolved in benzyl alcohol at increasing temperature, calculated by ^{1}H NMR. The water content is calculated based on the conversion of benzyl alcohol to dibenzyl ether and benzyl chloride.

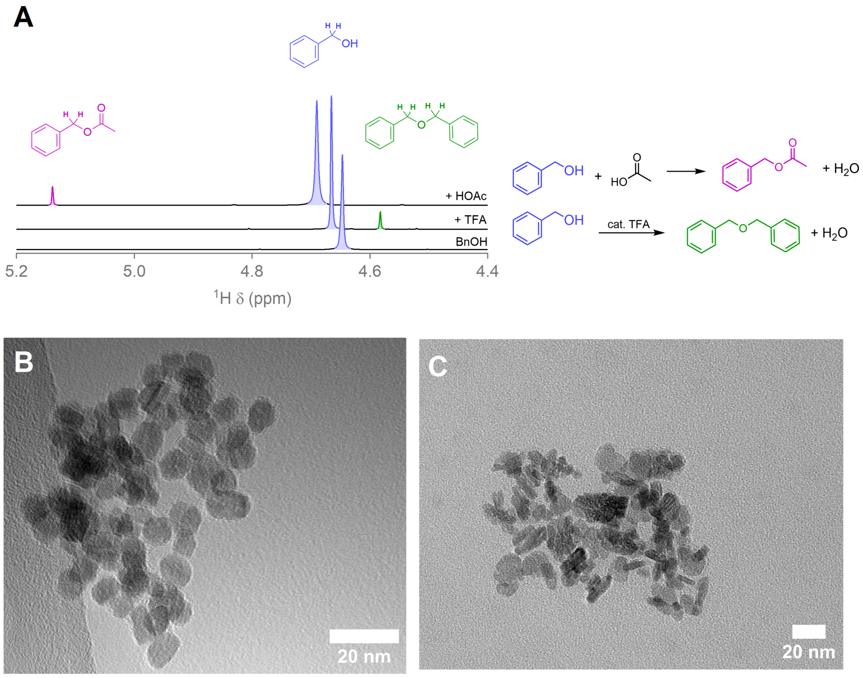

Figure S8: (A) ^{1}H NMR spectra of the reaction mixture supernatant after the synthesis of $Hf(OiPr)_4$.iPrOH (1) in benzyl alcohol and with additional (2) trifluoroacetic acid (TFA) and (3) acetic acid added. The resonances corresponding to benzylacetate, benzyl alcohol and dibenzylether are assigned. The spectra are shifted with a relative x-offset of 0.05 ppm for clarity. (B) TEM of the particles synthesized by the reaction of $Hf(OiPr)_4$.iPrOH and TFA in benzyl alcohol. (C) TEM of the particles synthesized by the reaction of $Hf(OtBu)_4$ and TFA in benzyl alcohol.

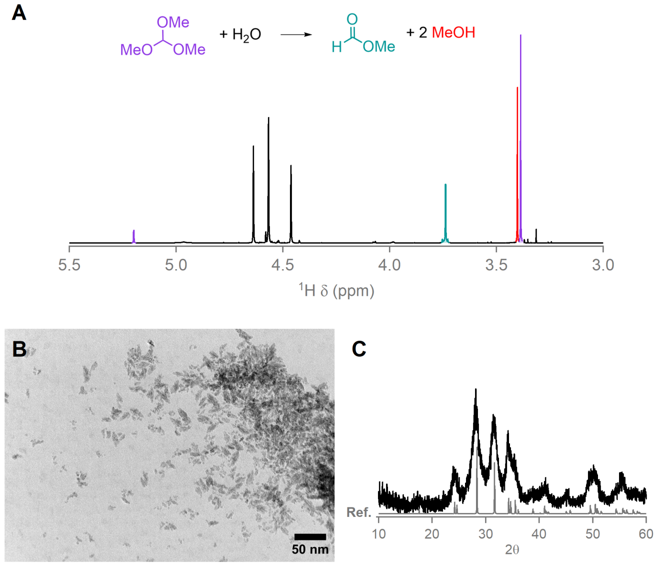

Figure S9: (A) ^1H NMR of the supernatant of the reaction mixture of $HfCl_4$.2THF in benzyl alcohol with trimethyl orthoformate (TMOF) added. Some unreacted TMOF is present, as well as the reaction products methyl orthoformate and methanol. The peaks between 4.5 and 4.7 ppm are the benzyl alcohol, benzyl chloride and dibenzyl ether peaks as previously determined. (B) TEM of synthesized NCs shows that their size is unaffected, but there is increased agglomeration. (C) Crystallinity is not affected. The grey reference spectrum is of monoclinic HfO_2.

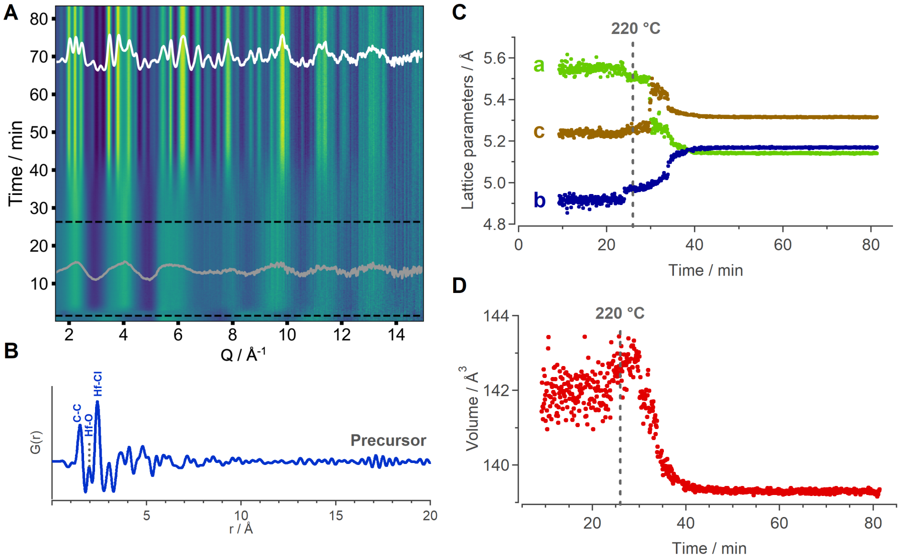

Figure S10: (A) Contour plot of the F(Q) with highlighted transitions and patterns plotted. (B) Complete PDF of the precursor structure. Sequential refinements showing the (C) lattice parameters and (D) volume as a function of time.

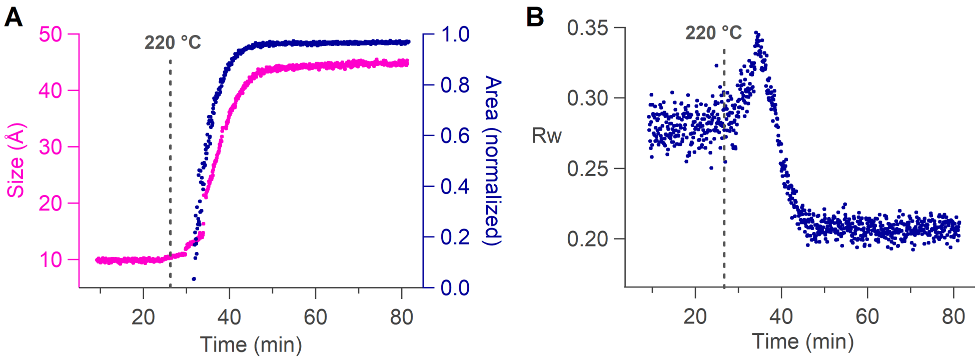

Figure S11: (A) The refined crystallite size (in real space) and the area of the (111) peak (in reciprocal space) as a function of time and (B) The R_w value, representing the quality of the fit in reciprocal space, as a function of time.

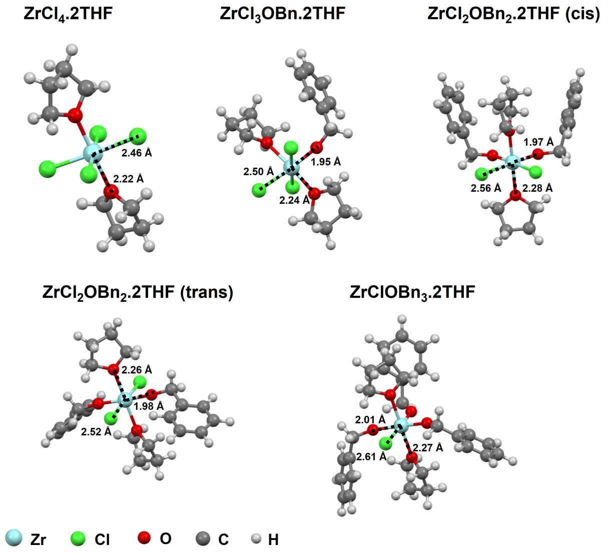

Figure S12: Possible precursor structures upon dissolving $ZrCl_4$.2THF in benzyl alcohol. The structures were calculated via DFT with increasing exchange of chlorides to benzyl alcoholates. Relevant bond lengths (Zr-O, Zr-Cl) are indicated.

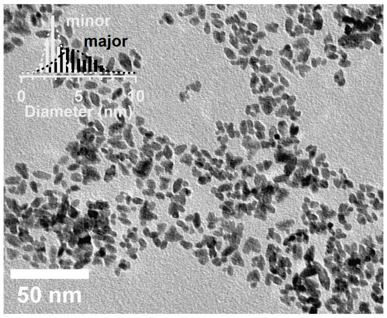

Figure S13: TEM image of the HfO_2 NCs extracted after 10 minutes at 220 °C. The size distribution is shown in the top left corner.

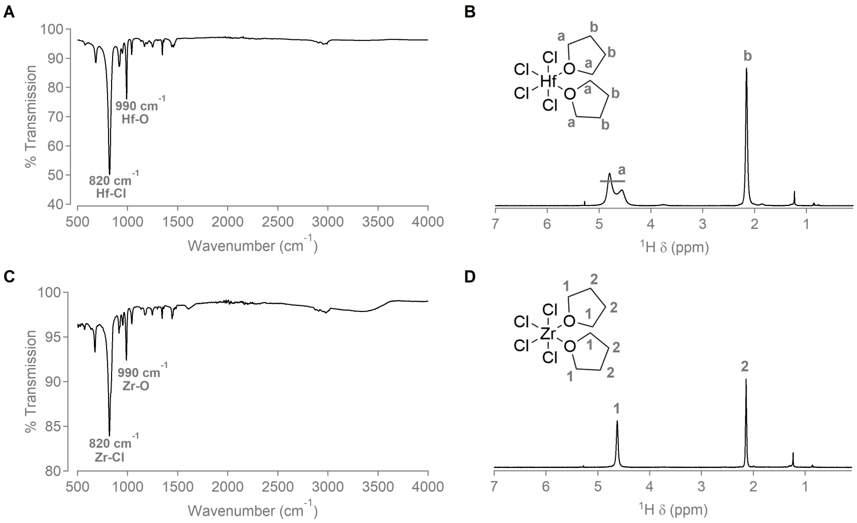

Figure S14: Characterization of the $HfCl_4$.2THF precursor via (A) FTIR and (B) ^{1}H NMR and of the $ZrCl_4$.2THF precursor via (C) FTIR and (D) ^{1}H NMR.

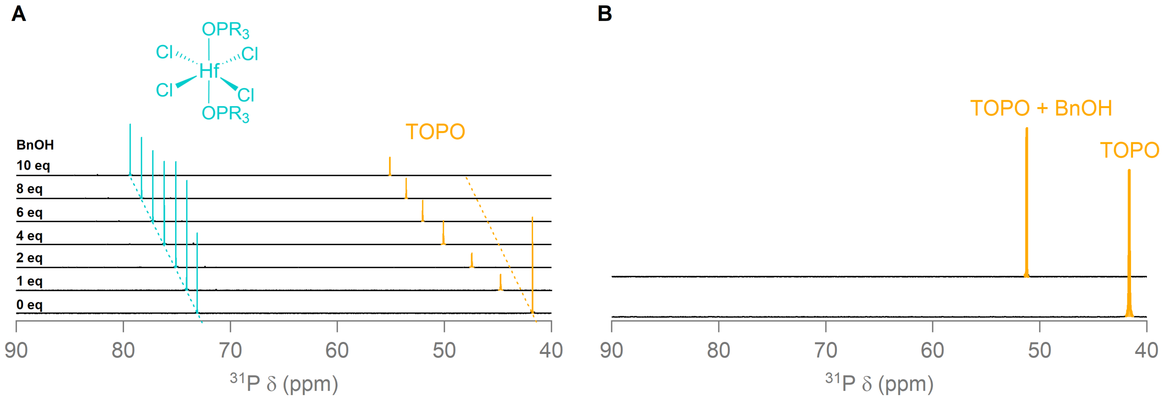

Figure S15: (A) ^{31}P NMR titration of $HfCl_4$.2THF with TOPO and BnOH without the vacuum step shows only the tetrachloride complex. (B) Shift of the free TOPO due to complexation with benzyl alcohol.

Tables

Table S1: Conversion (%) of benzyl alcohol (BnOH) in dibenzyl ether (BnOBn) and benzyl chloride (BnCl) as detected via quantitative ^{1}H NMR at different reaction times in the HfO_2 microwave-assisted synthesis. Each reaction was repeated at least three times to calculate the average and standard deviation. Ratios of the samples 5 min at 80 °C and 4 hours at 220 °C were calculated via peak deconvolution due to overlapping peaks.

Sample	% BnOH ($\mu \pm \delta$)	% BnOBn ($\mu \pm \delta$)	% BnCl ($\mu \pm \delta$)	% H_2O ($\mu \pm \delta$)
5 min at 80 °C	98.2 ± 0.5	1.0 ± 0.3	0.8 ± 0.2	1.3 ± 0.4
160 °C	86.8 ± 1.0	7.7 ± 0.6	5.5 ± 0.4	9.4 ± 0.7
180 °C	85.4 ± 0.6	8.8 ± 0.5	5.8 ± 0.2	10.2 ± 0.4
200 °C	83.8 ± 0.8	10.4 ± 0.7	5.8 ± 0.2	11.0 ± 0.6
1 min at 220 °C	75.6 ± 2.3	18.3 ± 2.0	6.1 ± 0.4	15.2 ± 1.4
3 min at 220 °C	68.1 ± 2.4	25.9 ± 2.3	6.1 ± 0.7	19.0 ± 1.9
5 min at 220 °C	59.8 ± 11.5	33.6 ± 11.1	6.6 ± 0.6	23.4 ± 6.1
10 min at 220 °C	42.0 ± 1.8	51.5 ± 1.7	6.5 ± 0.6	32.3 ± 1.4
30 min at 220 °C	28.8 ± 3.8	64.3 ± 3.3	6.9 ± 0.6	39.1 ± 2.2
2 h at 220 °C	22.8 ± 0.4	69.3 ± 1.4	7.9 ± 1.2	42.6 ± 1.9
4 h at 220 °C	21.4 ± 2.5	69.0 ± 3.5	9.6 ± 1.0	44.1 ± 2.7

Table S2: Fitting of the EXAFS data of Zr-O, Zr-Cl, Hf-O and Hf-Cl with single scatter paths.

Sample	Bond	$S_0{}^2$	N	σ^2	e_0	R
Zr [Zr]		0.900	12.459	0.00933	4.407	3.20377
$ZrCl_4$.2THF (25 °C)	Zr-O	0.900	2.674	0.00515	6.095	2.28045
$ZrCl_4$.2THF (25 °C)	Zr-Cl	0.900	4.060	0.00417	3.393	2.48520
$ZrCl_4$.2THF (100 °C)	Zr-O	0.900	2.624	0.00515	6.095	2.28262
$ZrCl_4$.2THF (100 °C)	Zr-Cl	0.900	3.945	0.00417	3.393	2.48578
$ZrCl_4$.2THF (140 °C)	Zr-O	0.900	3.151	0.00515	6.095	2.26256
$ZrCl_4$.2THF (140 °C)	Zr-Cl	0.900	3.220	0.00417	3.393	2.48962
$ZrCl_4$.2THF (160 °C)	Zr-O	0.900	4.721	0.00515	6.095	2.23664
$ZrCl_4$.2THF (160 °C)	Zr-Cl	0.900	1.389	0.00417	3.393	2.50844
HfO_2 [O]		0.900	2.000	0.00359	7.099	2.03640
HfO_2 [O]		0.900	3.000	0.00361	7.099	2.13660
HfO_2 [O]		0.900	2.000	0.00543	7.099	2.23240
HfO_2 [Hf]		0.900	1.848	0.00207	7.099	3.33505
HfO_2 [Hf]		0.900	4.056	0.00207	7.099	3.45000
$HfCl_4$.2THF (25 °C)	Hf-O	0.900	2.711	0.00757	8.854	2.30302
$HfCl_4$.2THF (25 °C)	Hf-Cl	0.900	5.060	0.00557	8.076	2.42654
$HfCl_4$.2THF (100 °C)	Hf-O	0.900	1.959	0.00757	8.854	2.27897
$HfCl_4$.2THF (100 °C)	Hf-Cl	0.900	4.449	0.00557	8.076	2.43764
$HfCl_4$.2THF (120 °C)	Hf-O	0.900	1.679	0.00757	8.854	2.22897
$HfCl_4$.2THF (120 °C)	Hf-Cl	0.900	3.952	0.00557	8.076	2.44334
$HfCl_4$.2THF (140 °C)	Hf-O	0.900	2.605	0.00757	8.854	2.13673
$HfCl_4$.2THF (140 °C)	Hf-Cl	0.900	3.029	0.00557	8.076	2.44739
$HfCl_4$.2THF (160 °C)	Hf-O	0.900	6.132	0.00757	8.854	2.12317
$HfCl_4$.2THF (160 °C)	Hf-Cl	0.900	0.672	0.00557	8.076	2.46524

Table S3: Parameters from real-space Rietveld refinement of the PDF data collected in the *in-situ* X-ray total scattering experiment. We use the monoclinic HfO_2 crystal structure with space group $P2_1/c$ as the structural starting model

Parameter	Initial	Final (81 min)	Intermediate (9 min)
R_w		0.22	0.42
Scale		0.503	0.515
Latt., a ($\AA$)	5.11	5.13	4.97
Latt., b ($\AA$)	5.17	5.18	5.18
Latt., c ($\AA$)	5.29	5.31	5.45
$U_{iso}(Hf)$(Å^2)		0.00692	0.00535
$U_{iso}(O)$(Å^2)		0.0716	0.239
Delta2 ($\AA^{-2}$)		3.73	4.69
Spdiameter, size ($\AA$)		45.8	10.9
Hf pos1	0.275	0.276	0.276
Hf pos2	0.460	0.461	0.465
Hf pos3	0.707	0.708	0.727

Table S4: Yield determination of HfO_2 NC syntheses at different reaction times.

Reaction time (h)	TGA (%)	Sample weight (-ligands) (mg)	Yield (%)
1 (normal)	82.80	75.10	44.5
1 (normal)	82.85	88.37	52.3
1 (normal)	79.46	91.70	54.4
2	80.41	115.39	68.4
2	82.82	123.31	73.1
2	82.82	125.80	74.5
3	80.89	118.09	70.0
3	82.82	121.49	72.0
3	82.82	134.22	79.5
4	82.25	137.27	81.4
4	82.82	123.23	73.0
4	82.82	139.80	82.8
5	82.86	124.37	73.8
5	82.82	135.65	80.4
5	82.82	128.38	76.1
1 (optimized)	83.91	141.98	84.2
1 (optimized)	86.61	143.51	85.0
1 (optimized)	82.24	133.15	78.8

Calculations

Equivalents of NMR measurements

As described in the experimental under 'Reaction aliquotes', aliquotes were taken from a solution consisting of 0.930 g (1 eq, 2 mmol) $HfCl_4$.2THF in 8 mL (38.468 eq, 76.936 mmol, 8.32 g) benzyl alcohol. NMR spectra were calibrated using the residual solvent peak as internal standard ($CDCl_3$: δH = 7.24 ppm). For each individual peak the baseline was manually corrected and the full peak was integrated. The % of each peak relative to each other was calculated based on the total area of all three peak intensities ($I_{\text{total}} = I_{\text{BnOH}} + I_{\text{BnCl}} + I_{\text{BnOBn}}$):

$$\%_{peak} = \frac{I_{peak}}{I_{total}} \tag{1}$$

Each equivalent is calculated as follows, taking into account that the peak assigned to dibenzyl ether corresponds to 4 protons and those assigned to benzyl alcohol and benzyl chloride correspond to 2 protons each:

$$Eq_{BnOH} = \%_{BnOH} \cdot 0.38468 \tag{2}$$

$$Eq_{BnCl} =: \%_{BnCl} \cdot 0.38468 \tag{3}$$

$$Eq_{BnBn} = \frac{\%_{BnOBn} \cdot 0.38468}{2} \tag{4}$$

Using the aforementioned assumption that all water created comes from the condensation of benzyl alcohol to dibenzyl ether and the reaction of benzyl alcohol with hydrogen chloride to benzyl chloride, the total equivalents of water created are:

$$Eq_{\text{H}_2\text{O}} = Eq_{BnOBn} + Eq_{BnCl} \tag{5}$$
